# Untargeted metabolomics confirms and extends the understanding of the impact of aminoimidazole carboxamide ribotide (AICAR) in the metabolic network of *Salmonella enterica*

**DOI:** 10.15698/mic2018.02.613

**Published:** 2017-11-22

**Authors:** Jannell V. Bazurto, Stephen P. Dearth, Eric D. Tague, Shawn R. Campagna, Diana M. Downs

**Affiliations:** 1 Department of Microbiology, University of Georgia, Athens, GA 30602.; 2 Department of Chemistry, University of Tennessee, Knoxville, TN 37996.

**Keywords:** aminoimidazole carboxamide ribotide (AICAR), cyclic AMP (cAMP), adenylate cyclase (CyaA), cyclic AMP phosphodiesterase (Icc), purine-histidine-thiamine (PHT) metabolic network, thiamine biosynthesis, untargeted metabolomics

## Abstract

In *Salmonella enterica*, aminoimidazole carboxamide ribotide (AICAR) is a purine biosynthetic intermediate and a substrate of the AICAR transformylase/IMP cyclohydrolase (PurH) enzyme. When *purH* is eliminated in an otherwise wild-type strain, AICAR accumulates and indirectly inhibits synthesis of the essential coenzyme thiamine pyrophosphate (TPP). In this study, untargeted metabolomics approaches were used to i) corroborate previously defined metabolite changes, ii) define the global consequences of AICAR accumulation and iii) investigate the metabolic effects of mutations that restore thiamine prototrophy to a *purH* mutant. The data showed that AICAR accumulation led to an increase in the global regulator cyclic AMP (cAMP) and that disrupting central carbon metabolism could decrease AICAR and/or cAMP to restore thiamine synthesis. A mutant (*icc*) blocked in cAMP degradation that accumulated cAMP but had wild-type levels of AICAR was used to identify changes in the *purH* metabolome that were a direct result of elevated cAMP. Data herein describe the use of metabolomics to identify the metabolic state of mutant strains and probe the underlying mechanisms used by AICAR to inhibit thiamine synthesis. The results obtained provide a cautionary tale of using metabolite concentrations as the only data to define the physiological state of a bacterial cell.

## INTRODUCTION

**Figure 1:**
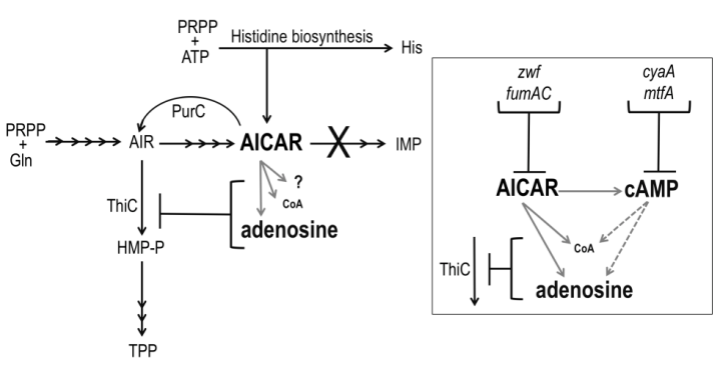
FIGURE 1: The role of AICAR in the purine histidine thiamine (PHT) metabolic network of *S. enterica*. Solid black arrows represent biochemical reactions; corresponding gene products are beside the reaction they catalyze. AICAR-mediated points of metabolic crosstalk that impact thiamine synthesis (ThiC activity) are indicated with gray arrows. *Inset:* AICAR accumulation leads to elevated cAMP. Elevated AICAR and cAMP independently decrease pantothenate (CoA) pools and increase adenosine pools demonstrating that these pools are subject to direct (solid lines) and indirect (dashed lines) effects of AICAR accumulation. These altered pools potentially contribute to decreased ThiC activity; loss of function mutations (*zwf*,* fumAC*,* cyaA*,* mtfA*) modulated AICAR and/or cAMP levels and restored thiamine synthesis.

In *Salmonella enterica*, investigating metabolic redundancy in the thiamine biosynthetic pathway is a well-established model system for identifying complex metabolic interactions 1,2. Studies with this system have characterized numerous connections between thiamine synthesis and diverse areas of metabolism including, amino acid biosynthesis 3,4,5,6, metabolite detoxification systems 5,7,8 and cofactor biosynthesis 9,10,11. By characterizing individual points of metabolic integration, these studies have expanded our understanding of the purine-histidine-thiamine (PHT) metabolic network and how it interacts with the metabolic network at large (Figure 1). Research with this model system has defined non-canonical roles of specific metabolites, elucidated catalytic mechanisms of biosynthetic enzymes and demonstrated the physiological relevance of redundant pathways in related organisms 12.

Aminoimidazole carboxamide ribotide (AICAR) is a prominent component of the PHT metabolic network and has an increasingly recognized, and complex, role within it (Figure 1). Though known primarily for being a purine biosynthetic intermediate, AICAR is also a histidine biosynthetic byproduct, a precursor to thiamine in some genetic contexts and an indirect inhibitor of thiamine synthesis. Thiamine pyrophosphate (TPP) is a nearly ubiquitous coenzyme that until recently was believed to be essential for central metabolic processes of all organisms 13. TPP is composed of separately synthesized thiazole and pyrimidine moieties. In bacteria and plants the pyrimidine moiety (4-amino-5-hydroxymethyl-2-methylpyrimidine, HMP) is synthesized in a pathway that branches off of the purine biosynthetic pathway. Although AICAR is synthesized beyond the last common intermediate of purine-HMP synthesis, aminoimidazole ribotide (AIR), it has long been known to abolish HMP synthesis when it accumulates intracellularly 14,15, and previous work has demonstrated that it indirectly inhibits the ThiC enzymatic step 16 by more than one mechanism 10 (Figure 1).

In *S. enterica,* mutants that accumulate AICAR have been generated by eliminating AICAR transformylase/IMP cyclohydrolase (PurH, EC:2.1.2.3/3.5.4.10), a bifunctional enzyme that catalyzes the last two steps in purine biosynthesis and the only known consumer of AICAR. Suppressor strains of the *purH* mutant that regained the ability to synthesize thiamine (HMP) were used to determine that AICAR (and its riboside derivative AICARs) is a direct inhibitor of pantoate-β-alanine ligase (PanC, EC:6.3.2.1). This inhibition decreased coenzyme A (CoA) levels to 33% of wild-type 10. CoA has an elusive role in HMP synthesis but data has shown that HMP synthase (ThiC, EC:4.1.99.17) is sensitive to decreased CoA levels *in vivo* 9,16 and suggest CoA contributes to the reducing environment of the cell 17 that may protect ThiC’s Fe-S cluster from oxidation 11,18. Further nutritional work suggested that AICAR had at least one additional negative impact on ThiC activity 10 and demonstrated that lesions in multiple genes involved in central carbon metabolism (*cyaA*, *gdhA*, *mtfA*, *zwf* and *fumAC*) could restore thiamine synthesis in a *purH* mutant 19. These data indicated that more than one metabolic pathway important for ThiC function was disrupted by AICAR accumulation and suggested that lesions in *cyaA*, *gdhA*, *mtfA*, *zwf* and *fumAC* modulated AICAR levels and/or neutralized its impact on ThiC activity.

Genetic and biochemical data from previous studies have defined numerous roles for AICAR in metabolism 10,20,21,22,23,24,25,26,27,28. The integration of the metabolic network suggested that AICAR accumulation would have a number of targets, each with their own downstream effects. Herein we investigate the global impact of elevated AICAR levels in *S. enterica* with untargeted metabolomics approaches. This study obtained metabolomics data that highlight the broad reach of AICAR in unexpected and distinct areas of metabolism. Importantly, this work demonstrates the potential, and describes caveats, of combining genetic analyses and metabolomics to deconstruct pleiotropic network perturbations in bacteria.

## RESULTS AND DISCUSSION

### Rationale for using global approaches to dissect the metabolic impact of AICAR

Extensive *in vivo* genetic and biochemical analyses have probed the structure of the metabolic network consisting of purine, histidine and thiamine biosynthetic pathways (PHT node) 3,10,16,29,30,31,32,33,34. While these approaches have provided significant metabolic and mechanistic insights, studies of the PHT system have reached the point where continued genetic analyses are laborious and unlikely to efficiently answer questions about metabolic network structure and integration. The PHT node was used as a test case for the implementation of metabolomics approaches to generate testable hypotheses to extend our understanding of the subtleties associated with metabolic integration.

An in-frame, non-polar deletion of *purH* in *S. enterica* lacks AICAR transformylase and IMP cyclohydrolase activities. In *S. enterica*, a *purH* mutant is auxotrophic for purines and thiamine, due to elimination of the last two purine biosynthetic reactions, and inhibitory effects of AICAR accumulation on ThiC, respectively 10,14,15. A number of nutritional supplements and suppressor mutations that restore thiamine-independent growth of a *purH *mutant have been described 10,19. Of specific interest was the additive effect of methionine and pantothenate, that demonstrated that the AICAR-mediated inhibition of pantothenate synthesis was not the only perturbation that constrained thiamine synthesis when AICAR accumulated 10. Null mutations in genes involved in pathways of central carbon metabolism alleviated constraints on thiamine synthesis 19 and added to the growing body of evidence that suggested that AICAR accumulation perturbed cellular homeostasis in a number of areas that then, directly or indirectly, compromised thiamine synthesis. The impact that AICAR accumulation has on cellular processes (in a range of organisms) such as purine synthesis, phosphate utilization, energy homeostasis (including gluconeogenesis and fatty acid synthesis) and one carbon metabolism has been highlighted with various studies 10,20,21,22,23,24,25,26,27,28.

### A *purH* mutation results in global metabolic changes

An untargeted metabolomics approach was used in a proof-of-principle experiment to analyze a strain lacking PurH. To validate the use of this approach in probing metabolism, it was critical that the metabolic features that have been described for *purH *mutants were captured in this experiment. The metabolome of a* purH* mutant was compared to that of the isogenic parental strain (wild-type) in different growth stages by taking samples at mid-log (Abs_650_ ~0.475), early stationary (Abs_650_ ~1.5) and late stationary (Abs_650_ ~1.4) phase. Reproducibility of stationary phase results was rigorously queried by repeating the same experiment on two sequential days and then months later on three sequential days. In each experiment, biological triplicates were used. Water-soluble metabolites were extracted from wild-type (DM10000) and *purH* mutant (DM12239) strains of *S. enterica* and analyzed via UPLC-MS, using slight modifications of previously established methods 35,36. The metabolomics data obtained from 15 samples (3 biological replicates x 5 experimental replicates) across stationary growth phases and 3 samples (3 biological replicates x 1 experiment) during exponential growth were used to identify metabolic changes that resulted when the PurH-catalyzed step in purine biosynthesis was blocked.

**Figure 2:**
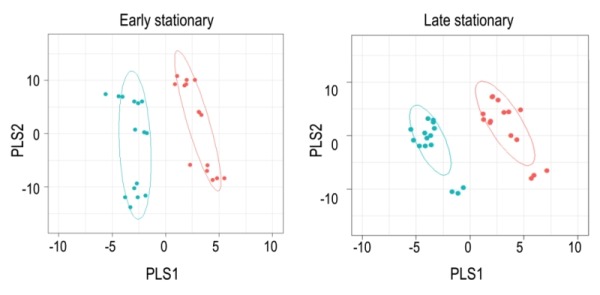
FIGURE 2: PLSDA of WT and *purH* mutant metabolomes. Untargeted metabolomics was performed on wild-type and *purH* mutant of *S. enterica* grown in glucose minimal medium supplemented with adenine and thiamine. Partial Least Square Discriminate Analysis (PLSDA) was used as a multivariate analysis tool to provide insight into the unique metabolic fingerprint of the *purH *mutant. Each dot represents one experimental replicate; WT is in cyan, the* purH* mutant is in peach. Confidence intervals are shown by the colored ellipses.

Using Metabolomic Analysis and Visualization Engine (MAVEN) software developed by Clasquin *et al*., approximately 140-180 metabolites were identified in each experiment 37. Both multi- and single-variate approaches were used to analyze the data. Multivariate analysis using Partial Least Square Discriminate Analysis (PLSDA) resulted in the visual separation of the metabolomes in multidimensional space, demonstrating clearly that the metabolite profiles of a *purH *mutant are considerably different when compared to those of wild-type (Figure 2, Table 1). In early stationary phase, 22 metabolites were determined to be significant drivers of class separation, and 23 were significant in both late stationary and exponential phase. Five of these metabolites were common among the three growth phases, supporting the conclusion that these differences are linked to genotype not environmental factors. In particular, the class separation was conserved despite replicate experiments being done months apart. Table 1 shows all the variable importance in projections (VIP) scores >1 from each PLSDA. Over half of the metabolites with a VIP of >1 are present in at least two of the growth stages. Importantly, AICAR was the predominant driver of separation (VIP=3-7). This result was gratifying in that it reflected the major change expected in a *purH* mutant that lacks the AICAR transformylase enzyme. The data further showed that metabolites not directly related to the *purH* mutation contributed to the separation of the metabolomes.

**Table 1:** Important metabolites as determined by PLSDA. Variable importance in projections (VIP) scores for the three growth phases were tested. Metabolites that have VIP score greater than 1 are indicated. Metabolites with VIPs>1 in 2 growth phases are bolded; those with VIPs>1 in 3 growth phases are bolded and boxed. The metabolites denoted as Glu^2 ^and Malate^2^ are isomers of the listed compound and have an undetermined structure.

**Exponential**		**Early Stationary**		**Late Stationary**	
**Metabolite**	**VIP**	**Metabolite**	**VIP**	**Metabolite**	**VIP**
**AICAR**	7.3736	**Acetyl-P**	1.5362	**Acetyl-P**	1.4554
**AICARs**	3.1681	**AICAR**	6.5634	Aconitate	1.3504
ATP	1.2336	**AICARs**	1.4952	**AICAR**	3.0974
**Betaine/Val**	3.5394	**Asp**	1.5125	**Asp**	1.7768
Deoxyribose-P	1.2292	**Betaine/Val**	5.5507	**Betaine/Val**	7.2269
**2,3-Dihydroxybenzoate**	1.6171	**Citrate**	2.5485	**Citrate**	3.8841
Homoserine/Thr	1.0629	**2,3-Dihydroxybenzoate**	2.2062	Cyclic AMP	1.0003
**2-Hydroxy-2-methylbutanedioic acid**	1.3098	**Glu^1^**	2.1229	Deoxyinosine	1.3386
**Isopropylmalate**	3.9679	Glu^2^	1.5235	Dimethylglycine	3.1393
**Ketoisovalerate**	1.2343	**Hydroxyisocaproate**	1.4819	**Glu^1^**	3.4531
**Malate^1^**	1.1813	**Isopropylmalate**	3.3397	Gln	1.0348
**Malate^2^**	1.1813	Leu/Ile	1.5560	Glycerate	1.4783
**Methylmalonate**	3.1222	**Malate^1^**	3.6822	**Hydroxyisocaproate**	1.8499
**N-acetylglutamate**	4.9175	**Malate^2^**	3.0078	**2-Hydroxy-2-methylbutanedioic acid**	1.9445
NADH	1.1018	**Methylmalonate**	2.2483	Hypoxanthine	1.5394
**N-carbamoyl-L-Asp**	1.1942	**N-acetylornithine**	2.0604	**Isopropylmalate**	2.6574
**Orotate**	1.4249	**N-carbamoyl-L-Asp**	1.3100	**Ketoisovalerate**	1.3586
6-Phosphogluconate	1.5895	**Orotate**	1.3731	Lipoate	1.7865
3-Phosphoglycerate	1.2241	**Pantothenate**	2.0429	**Malate^1^**	2.9740
**Pyroglutamate**	1.5009	**Pyroglutamic acid**	1.1308	**Malate^2^**	2.6831
Ser	1.2722	Pyrophosphate	1.2704	**Pantothenate**	2.8073
UDP-D-glucose	1.5608	UDP-glucose	1.4248	Phe	1.1058
UDP-N-acetyl-glucosamine	1.0165			Pyroglutamic acid	1.0548

**Figure 3:**
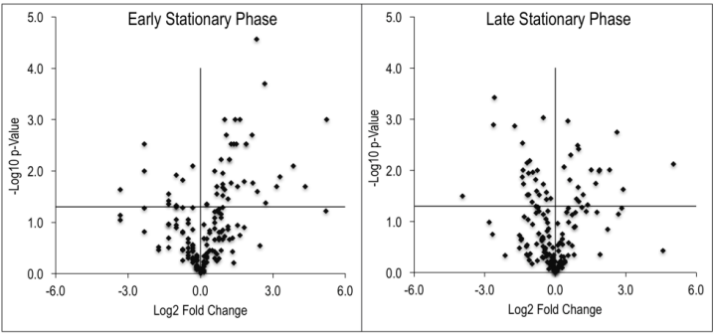
FIGURE 3: The metabolome of a *purH* mutant is significantly altered. Untargeted metabolomics was performed on wild-type and *purH* mutant of *S. enterica* grown in glucose minimal medium supplemented with adenine and thiamine. Relative concentrations of metabolites were determined by calculating the ratio of the average MS ion intensities (peak areas) in wild-type and *purH* strains (*purH*/WT). Here the log transformed fold changes (Log2) and p-values (-Log10) were plotted against each other, distributing the metabolite pool decreases and increases to the left and right of the x=0 point, respectively. The horizontal line (y=1.3) indicates the threshold above which p-values were considered statistically significant (<0.05). These data are derived from one representative experiment (Table S2).

**Figure 4:**
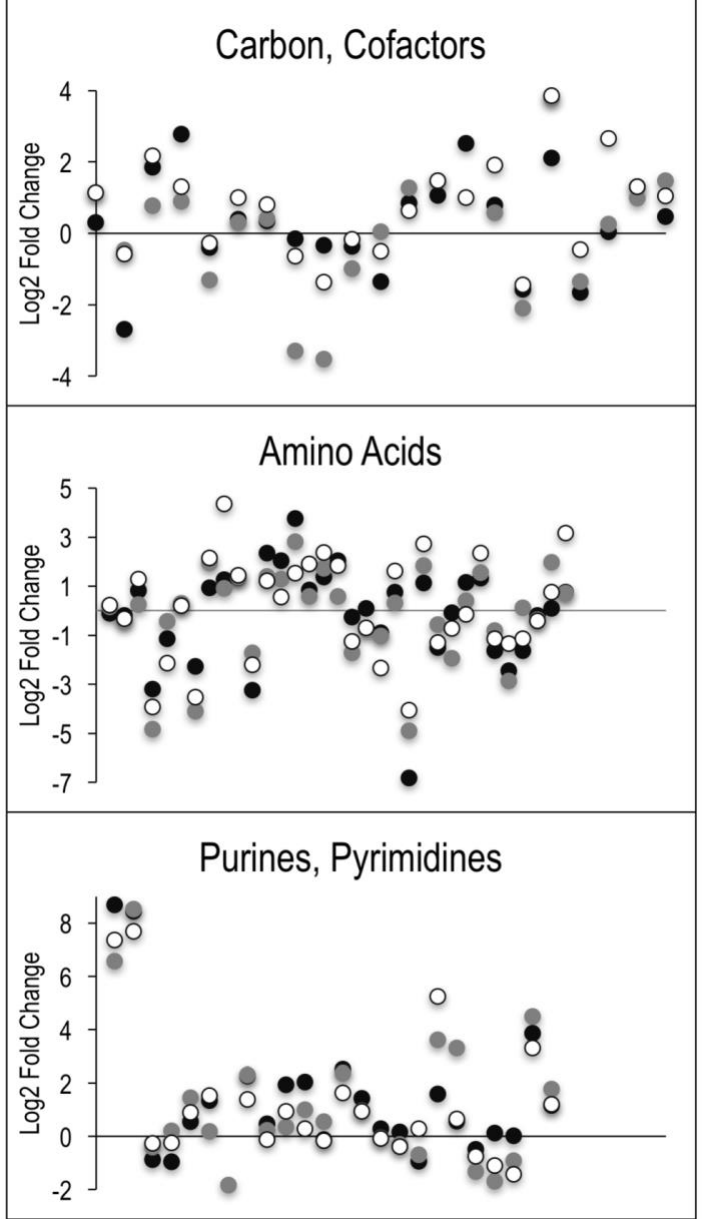
FIGURE 4: AICAR perturbs various areas of metabolism. Untargeted metabolomics was performed on wild-type and *purH* mutant of *S. enterica* grown in glucose minimal medium supplemented with adenine and thiamine. Relative concentrations of metabolites were determined by calculating the ratio of the average MS ion intensities (peak areas) in wild-type and *purH* strains (*purH*/WT). Here the log transformed fold changes (Log2) of metabolites with statistically significant differences were plotted to distribute the decreases and increases (below and above) the y-axis. Each point along the x-axis represents a metabolite. Each of 3 experimental replicates are differentiated by color. Specific metabolites, fold changes and p-values are provided in Table S3.

Despite the clear separation of the metabolite profiles between strains (Figure 2, Table 1), there were variations in the detection of some metabolites between experiments. Most notably, the second set of experiments (3 sequential days) consistently detected ~20% more metabolites than the first set of experiments (2 sequential days). The few differences in detection between growth phases could reasonably reflect physiological fluctuations that decreased metabolite concentrations below the limit of detection, and thus these were not concerning. In all cases, the parameter of interest was the ratio of metabolite concentrations (pool sizes) present in the wild-type vs. *purH* mutant strains; no attempts were made to assess the absolute concentrations of metabolites. The distribution of calculated fold changes (*purH*/WT) and the relative abundance of statistically significant differences (as determined by p-value from an unpaired Student’s t-test) observed in a given experiment are shown in Figure 3. Here, each data point is the ratio for one metabolite and the horizontal line represents the threshold above which p-values indicated a significant difference (<0.05). These data suggested that a large number of metabolites are altered in a *purH* mutant; this result was surprising since bacteria are proficient at restoring cellular homeostasis after a perturbation 38,39,40. In each experiment, across all growth phases, 25-30% of the detected metabolites were differentially present in wild-type and *purH* mutant strains. In fact, this may be an under estimate since this number represents only metabo-lites where statistical significance of a non-one ratio could be clearly assigned. To distill true changes in the metabolome, statistically significant changes identified in early and late stationary phases were compared across 3 experiments (three sequential days). When fold-change trends were maintained across experiments, these changes were considered experimentally validated (Table S3). These data demonstrated that several metabolite pools involved in carbon, cofactor, amino acid, purine and pyrimidine metabolism were altered in a *purH* mutant and indicated the range of cellular processes that were perturbed by AICAR accumulation (Figure 4, Table S3). The variation between the relative levels of metabolites between experiments suggested that untargeted metabolomics was not appropriate as a stand-alone approach to quantify all metabolic differences between strains at the level of replication presented herein, but valuable information on key drivers of metabolic differentiation between the strains were detected. Fewer reports exist of metabolomics investigations in bacteria than in other organisms, and this observation coupled with these data may highlight difficulties in performing metabolomics in these microorganisms due to the coupled biological and technical variability when measuring bacterial metabolites.

### Untargeted metabolomics captures known metabolic features of a *purH* mutant

Beyond the overall differences between the *purH *and wild-type metabolomes depicted in Figures 2-4, the datasets were culled for evidence that they reflected specific prior biological knowledge. In this vein, key metabolites expected to be different in a *purH *mutant (AICAR, AICARs, pantothenate, adenosine) were evaluated in the untargeted metabolomics datasets (Table 2) 10,14,15,20. In every sample, across experiments and growth phases, AICAR pools were considerably higher in the *purH* mutant and the data were statistically significant in 5 of 5 early stationary phase and 2 of 5 late stationary phase samples. Gratifyingly, the ratio of AICAR in a *purH* mutant relative to wild-type during mid-log phase (65.0-fold, p-value 0.007) was on par with a previous report where investigators showed a 10-fold increase in AICAR levels within just an hour of anti-folate (PurH inhibition) treatment 41. The AICAR riboside (AICARs) is the predominant form of AICAR that accumulates in a *purH* mutant 42. In two experiments AICARs was not detected in wild-type or *purH* samples. In the remaining 3 experiments, however, the expected accumulation of AICARs was observed. Although all experiments showed a >90-fold increase of AICARs in *purH *vs. wild-type in early stationary phase, statistical analysis attributed significance to the values in just 1 of 3 the experimental replicates. This result indicated that not all expected metabolic changes would be identified by our methods of data analyses, even in the face of gross metabolite changes. A point of concern was the inability to verify the statistical significance of the data with multiple experimental repetitions.

**Table 2:** Metabolomics captures known metabolic changes of a *purH *mutant. Untargeted metabolomics was performed on WT and Δ*purH* mutant of *S. enterica* grown in glucose minimal medium supplemented with adenine and thiamine. Relative concentrations of 3 key metabolites whose physiological changes have been previously described were extracted. ^a ^Experimental replicates consisted of 3 biological replicates of each strain. ^b^ Fold changes were the ratio of the average MS ion intensities (peak areas) in WT and Δ*purH* strains (Δ*purH*/WT). ^c^ p-values indicative of statistically significant differences are noted as follows <0.05 (**), <0.01 (***).

		**Early stationary**			**Late stationary**	
	**Exp.^a^**	**Fold Change^b^**	**p-value^c^**		**Fold Change**	**p-value**
AICAR	1	346.6	0.009***		16.8	0.116 -
	2	367.2	0.003***		34.1	0.032**
	3	206.0	0.017**		32.7	0.007***
	4	124.9	0.020**		82.0	0.053 -
	5	133.0	0.033**		47.9	0.055 -
						
AICARs	1	407.2	0.194 -		2.8	0.011**
	2	94.4	0.027**		3.4	0.395 -
	3	164.3	0.079 -		7.4	0.023**
	4	ND			ND	
	5	ND			ND	
						
Adenosine	1	1.4	0.568 -		1.1	0.755 -
	2	2.7	0.237 -		1.3	0.442 -
	3	1.8	0.006***		1.5	0.055 -
	4	1.4	0.093 -		0.7	0.164 -
	5	1.2	0.098 -		0.8	0.273 -
						
Pant	1	0.3	0.004***		0.8	0.607 -
	2	0.2	0.002***		0.2	0.0001***
	3	0.4	0.038**		0.2	0.0004***
	4	ND			ND	
	5	ND			ND	

Data in Table 2 showed there was a dramatic decrease (~10-fold) in AICAR and AICARs accumulation in the *purH *mutant in late stationary phase. Analyses of the culture supernatants in late stationary phase showed an increase in AICAR in the* purH* vs wild-type supernatants (53.9-fold, p-value 0.002). Together these data suggested AICAR and AICARs were secreted in late stationary phase, consistent with a previous report of the accumulation of AICAR/AICARs in culture medium of the *purH* mutants 15,42.

Genetic analyses led to the finding that during exponential growth total CoA levels in a *purH* mutant were decreased to 33% of wild-type, due to inhibition of a pantothenate biosynthetic enzyme by AICAR 10. Although CoA was not detected by the metabolomics platform used here, pantothenate was reliably detected and consistently present at lower levels in a *purH *mutant (Table 2). Although pantothenate was only detected in 3 of 5 experiments, in these replicate experiments the pantothenate levels averaged 30% of wild-type during early stationary phase (3 of 3, p-value <0.05) and 40% during late stationary phase (2 of 3, p-value <0.05) and were thus well-correlated with altered CoA levels.

Finally, during growth on adenine (as cells are grown here) adenosine pools are expected to be inflated in a *purH* mutant as a result of direct inhibition of adenosine deaminase by AICAR 20. Increased adenosine pools in a *purH* mutant were captured by the metabolomics platform in all data from early stationary phase (avg. ~1.7-fold increase) with modest statistical significance (Table 2). Recently, adenosine was identified as a direct inhibitor of ThiC 43, thus these elevated pools may further constrain thiamine synthesis in a *purH* mutant. These data supported the ability of untargeted metabolomics to detect relevant metabolite changes, and again emphasized the problematic issue of statistical significance that was often lacking despite clear non-one ratios between the strains.

On several occasions our analyses failed to assign statistical significance to changes that were known to occur. In some instances, such as AICAR accumulation in a *purH* mutant in late stationary phase, slightly more lenient p-values would have resolved this (Table 2). In other cases, such as AICARs and adenosine (Table 2), p-values were exceedingly high and replicate experiments were critical in identifying a trend with confidence. Herein we chose to be conservative and prioritized eliminating false positives with a stringent p-value cut off. However, implementing a fold change cutoff (e.g., >2.0), in addition to the t-test, might more completely capture relevant changes 44.

A false positive discovery rate of statistically significant changes was evaluated by comparing the *purH* mutant used throughout this study to an isogenic strain that contained a neutral transposon insertion in two sequential experiments (3 biological replicates per experiment). The number of statistical significant differences between these presumably identical strains were 3.6 and 12.1% of those found in the same experiments when the *purH* mutant was compared to wild-type (data not shown). When these differences were further evaluated to see if their respective trend held up in the second experiment, half of them did not.

In total, the data in Table 2 included metabolites that had both small (pantothenate, adenosine) and large (AICAR, AICARs) fold changes in a *purH *mutant compared to wild-type. Significantly, these metabolites were biologically relevant, and each had been shown to differ between a *purH *mutant and wild-type in experiments that used traditional reductionist approaches. Thus, while some caveats with the untargeted metabolomics approach were raised, the data were consistent with previous knowledge and provided confidence in the metabolite trends detected with an untargeted metabolomics approach.

### Metabolomics patterns suggest a metabolic explanation for suppressor mutations

**Figure 5:**
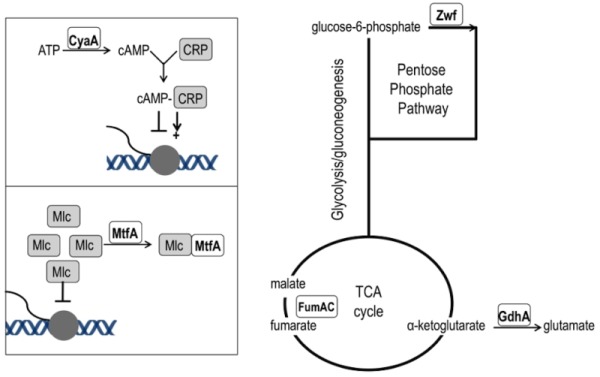
FIGURE 5: Network position of *purH* suppressor mutations. Loss-of-function mutations that suppress the thiamine requirement of a *purH* mutant have a role in various aspects of central carbon metabolism. The *cyaA *gene encodes adenylate cyclase and its product (cAMP) is the effector of the CRP transcriptional regulator that modulates >100 genes 45. The* mtfA* gene encodes the Mlc titration factor; Mlc is a global regulator of sugar metabolism. Both cAMP-CRP and MtfA are part of the PTS signal transduction pathway 46. Fumarate dehydratase (*fumAC*) and glutamate dehydrogenase (*gdhA*) function in, or just peripheral to, the TCA cycle. Glucose-6-phosphate dehydrogenase (*zwf*) is the first enzyme of the oxidative pentose phosphate pathway.

Data from extensive *in vivo *experimentation determined that the accumulation of AICAR is responsible for the inability of a *purH *mutant to synthesize thiamine. We previously reported the isolation of loss-of-function mutations that restored thiamine synthesis in a *purH* mutant. The relevant loci (*gdhA*, *mtfA*, *zwf* and *fumAC*) are involved in central metabolic processes, and an explanation for their ability to restore thiamine synthesis was not immediately apparent 19 (Figure 5). A metabolomics approach was used to query the relevant metabolites in the suppressor strains compared to the parental *purH *mutant. When all experiments were considered, there was a trend indicating that some suppressor strains had less AICAR/AICARs than the parental *purH *mutant. Unfortunately, AICARs was not detected in 1 of the 2 experiments, and the conclusion about decreased pools was typically supported with statistically significant data in only one of the two experiments for each mutant. With these caveats, the metabolomics data indicated that lesions in *mtfA*, *zwf* and *fumAC* appeared to decrease AICAR/AICARs concentrations to approximately half of that in the *purH *parent strain (Table 3). Thus, while they failed to provide any mechanistic information, these data were consistent with the fact that increased AICAR prevented thiamine synthesis in a *purH *mutant.

Significantly, pantothenate was increased in the* zwf* and *fumAC* mutants in late stationary phase samples, which suggested that pantothenate synthesis was partially restored by the decrease in AICAR caused by *zwf *and *fumAC* mutations. These data were consistent with lowered pantothenate being a downstream consequence of AICAR accumulation. Despite the generally appropriate trend, these data did not answer the question of whether, and how, such an apparently modest increase in pantothenate synthesis could increase the efficiency of the ThiC-catalyzed step in thiamine synthesis. In contrast*,* the *mtfA* mutation decreased the AICAR/AICARs levels in the *purH *mutant, while also generating a further 10-fold decrease (compared to *purH* mutant) in pantothenate levels in early stationary phase (p-values 0.075, 0.001) and unchanged pantothenate levels in late stationary phase. These results were unexpected since they were not obviously consistent with the *zwf* and *fumAC* suppressor mutations that lower AICAR/AICARs. Whether this finding is due to a technical artifact, or predicts that MtfA modulates pantothenate levels will have to be explored experimentally. In contrast to all other suppressor mutations, AICAR, AICARs and pantothenate were not statistically changed by a *gdhA* muta-tion in any of the experiments and statistically significant changes in the respective metabolome were not obviously integrated with thiamine synthesis. Collectively these data suggest that *zwf*, *fumAC* and *mtfA *suppressor mutations decrease AICAR/AICARs and restore thiamine synthesis via an effect downstream of pantothenate synthesis or by a mechanism unrelated to CoA. These data also suggest that the *gdhA* mutant employs a mechanism that circumvents elevated levels of AICAR present in a *purH* mutant. The complexity of the metabolomics data, while looking at only 3 metabolites, emphasizes the difficulty in deciphering the network connections without a phenotypic context. If the qualitative trends observed are significant, they suggest multiple paths to suppression exist. With confidence in the techniques used to generate them, the data facilitate the development of hypotheses that can be tested experimentally to extend our understanding of the metabolic system.

**Table 3:** Changes in central carbon metabolism reverse consequences of *purH* deletion. Metabolite fold changes were quantified in untargeted metabolomics analyses of WT and mutant strains of *S. enterica*. Cells were grown in glucose minimal medium supplemented with adenine and thiamine and harvested during early (ES) and late stationary (LS) phase growth phases in duplicate experiments, each consisting of 3 biological replicates. ^a^ Fold changes were the ratio of the average MS ion intensities in WT and Δ*purH* strains (Δ*purH*/WT ). ^b^ Fold changes were the ratio of the average MS ion intensities in Δ*purH* and Δ*purH* double mutants (Δ*purH mutX*/ Δ*purH).* ^c^ p-values indicative of statistically significant differences are denoted by: **(<0.05), ***(≪0.01).

**Genotype**	**AICAR**				**AICARs**				**Pant**			
	**ES1**	**ES2**	**LS1**	**LS2**	**ES1**	**ES2**	**LS1**	**LS2**	**ES1**	**ES2**	**LS1**	**LS2**
Δ*purH^a^*	367.2***	206.0**	34.1**	32.7***	ND	169.1	ND	7.6**	0.2***	0.4**	0.2***	0.2***
Δ*purH gdhA^b^*	0.8	1.8	1.4	0.8	ND	1.4	ND	0.7	0.7	0.3	1.0	1.3
Δ*purH mtfA^b^*	0.7	0.6	0.6	0.4**	ND	0.8	ND	0.1**	0.1	0.1***	0.8	1.0
Δ*purH zwf^b^*	0.5	0.6	1.1	0.7	ND	0.1	ND	0.3**	0.8	1.7	1.4	4.7**
Δ*purH fumAC^b^*	0.4***	0.6	0.5	0.5**	ND	0.5	ND	0.2**	1.1	1.1	3.2***	5.7***

### cAMP accumulates in a *purH* mutant and inhibits thiamine synthesis

Thiamine synthesis was restored in a *purH* mutant by a lesion in *cyaA* (encoding adenylate cyclase) 19. When exogenous cAMP, the product of adenylate cyclase, was added to the medium the *purH cyaA* mutant strain again required thiamine (data not shown). These data suggested a model in which i) cAMP levels were elevated in a *purH* mutant and ii) the increased cAMP contributed to the inability of a *purH *mutant to generate sufficient thiamine for growth. Thus it was gratifying that the metabolomics data showed a 2.2-fold (avg. of statistically significant values) increase in cAMP levels in a *purH *mutant over those in wild-type (Table 4). Elevated cAMP in a *purH *mutant versus wild-type strain was independently verified using an ELISA assay, which detected a 2.1-fold difference (p-value 0.004). These data supported a model where AICAR accumulation leads to elevated cAMP levels, which in turn modulates cellular metabolism, including the inhibition of thiamine biosynthesis.

**Table 4:** cAMP accumulates in a Δ*purH* mutant. cAMP levels were measured in WT and mutant strains of *S. enterica* by untargeted metabolomics (relative levels) or by a competitive enzyme-linked immunosorbent assay (ELISA). Cells were grown in minimal medium containing glucose, adenine and thiamine medium and harvested during early (ES) and/or late stationary (LS) phase (only harvested during ES for ELISA). Each experiment consisting of 3 biological replicates of each strain. Fold changes were the ratio of the average MS ion intensities between two strains. ^a^ p-values indicative of statistically significant differences are denoted by: **(<0.05), ***(<0.01).

	**Untargeted metabolomics^a^**				**ELISA (pmol/mL)**		
**Genotype**	**ES1**	**ES2**	**LS1**	**LS2**	**Rep**	**WT**	**Δ*purH***
	Fold change (*mutX*/WT)						
Δ*purH*	2.2	2.2**	1.3	1.6***	1	28.5	57.0
Δ*icc*	2.7	3.3***	1.5	2.2	2	23.7	48.7
	Fold change (Δ*purH mutX*/Δ*purH*)				3	25.6	58.6
Δ*purH gdhA*	0.9	0.5	1.1	0.7	**Avg.**	**25.9**	**54.7**
Δ*purH mtfA*	0.1	0.3***	1.0	0.7***			
Δ*purH zwf*	0.4	0.3***	1.4**	0.4***	Fold Change		2.1
Δ*purH fumAC*	0.8	0.6**	0.5***	0.8	p-value		0.004***

### Identification of downstream effects of elevated cAMP

An insertion deletion of the *icc* locus was made in an otherwise wild-type *S. enterica* strain to isolate the global effects of increased cAMP from those caused by increased AICAR levels. The *icc* mutant strain lacks cAMP phosphodiesterase activity and thus cannot catalytically degrade cAMP. In replicate experiments, elevated cAMP levels were observed in the untargeted metabolomics dataset of an *icc *mutant in early (3.0-fold) and late (1.9-fold) stationary phases. Importantly, the AICAR/AICARs pools in the *icc *mutant were unchanged from wild-type, which allowed us to decouple global changes arising from elevated cAMP versus AICAR. Changes in the metabolome that resulted from the 2- to 3-fold increase in cAMP in the *icc* mutant were compared to changes present in a *purH *mutant. Several metabolites had similar trends in the *purH* and *icc* mutants, but it was particularly notable that their pantothenate pools were comparably reduced compared to wild-type (Table 5). AICAR and AICARs are direct inhibitors of the pantothenate biosynthetic enzyme PanC, which is presumed to be responsible for the decreased pantothenate (CoA) in the *purH *mutant. These data indicated the mechanism of decreased pantothenate differed in the two strains and suggested that pantothenate synthesis is compromised on multiple fronts (direct inhibition, regulatory modulation) when AICAR accumulates.

**Table 5:** Increased cAMP levels account for a subset of perturbations observed in a Δ*purH* mutant.

	**Early Stationary Phase**								**Late Stationary Phase**							
	**Exp 1**				**Exp 2**				**Exp 1**				**Exp 2**			
	Δ*purH*/WT		Δ*icc*/WT		Δ*purH*/WT		Δ*icc*/WT		Δ*purH*/WT		Δ*icc*/WT		Δ*purH*/WT		Δ*icc*/WT	
	**FC**	p-val	**FC**	p-val	**FC**	p-val	**FC**	p-val	**FC**	p-val	**FC**	p-val	**FC**	p-val	**FC**	p-val
cAMP	**2.2**	0.165	**2.7**	0.293	**2.2**	0.035	**3.3**	0.004	**1.3**	0.105	**1.5**	0.184	**1.6**	0.005	**2.2**	0.098
Pantothenate	**0.2**	0.002	**0.6**	0.367	**0.4**	0.038	**0.2**	0.008	**0.2**	0.000	**0.2**	0.000	**0.2**	0.000	**0.6**	0.128
Isopropylmalate	**0.1**	0.005	**0.4**	0.080	**0.1**	0.073	**0.2**	0.098	**4.1**	0.002	**0.4**	0.008	**0.6**	0.068	**0.4**	0.063
Phosphoserine	**0.0**	0.030	**0.4**	0.079	**0.1**	0.023	**0.4**	0.033	**5.1**	0.048	**0.7**	0.376	**1.1**	0.756	**0.7**	0.568
Adenine	**0.7**	0.149	**0.4**	0.014	**0.8**	0.287	**0.7**	0.338	**1.5**	0.038	**0.2**	0.011	**1.2**	0.588	**3.2**	0.339
CMP	**0.4**	0.061	**0.3**	0.033	**0.6**	0.015	**1.6**	0.284	**0.5**	0.016	**0.5**	0.028	**0.6**	0.063	**0.3**	0.008
dCMP	**6.1**	0.025	**1.9**	0.336	**3.1**	0.021	**0.9**	0.834	**1.3**	0.036	**1.7**	0.347	**1.2**	0.693	**0.3**	0.100
dUMP	**22.2**	0.048	**2.7**	0.180	**8.9**	0.014	**1.0**	0.968	**1.1**	0.843	**1.5**	0.208	**1.1**	0.721	**1.6**	0.181
DHAP	**1.7**	0.144	**0.5**	0.147	**4.5**	0.017	**1.7**	0.105	**2.3**	0.031	**4.4**	0.208	**1.7**	0.129	**1.9**	0.332
UDP-Glucose	**13.2**	0.086	**3.9**	0.239	**2.0**	0.189	**3.0**	0.107	**3.0**	0.050	**14.0**	0.002	**0.5**	0.428	**5.0**	0.198
Ribose 5-P	**5.0**	0.124	**1.1**	0.780	**1.7**	0.033	**1.4**	0.198	**2.1**	0.078	**3.8**	0.142	**1.5**	0.103	**1.4**	0.417
Hydroxyisocaproate	**1.9**	0.314	**0.9**	0.743	**20.2**	0.020	**1.3**	0.155	**1.3**	0.089	**1.7**	0.263	**6.2**	0.002	**7.6**	0.299
Carbamoyl-P	**3.5**	0.155	**1.3**	0.628	**6.5**	0.042	**2.0**	0.307	**0.5**	0.330	**2.3**	0.086	**0.4**	0.091	**1.6**	0.097
Glu	**1.2**	0.811	**1.1**	0.909	**2.4**	0.003	**4.8**	0.051	**1.2**	0.785	**1.8**	0.040	**0.9**	0.518	**0.9**	0.647
Met	**0.5**	0.001	**0.2**	0.015	**0.2**	0.053	**0.3**	0.063	**1.3**	0.609	**0.6**	0.408	**0.6**	0.052	**1.0**	0.837
Aminoadipate	**2.5**	0.114	**1.1**	0.837	**2.7**	0.001	**1.4**	0.164	**1.8**	0.005	**2.2**	0.012	**2.0**	0.021	**1.0**	0.753
Phenylpyruvate	**3.1**	0.155	**4.2**	0.038	**2.3**	0.079	**2.9**	0.309	**1.2**	0.594	**1.4**	0.186	**1.2**	0.823	**1.6**	0.451
Phe	**6.9**	0.083	**3.0**	0.226	**2.9**	0.020	**8.7**	0.314	**0.7**	0.018	**1.7**	0.069	**0.8**	0.276	**0.7**	0.200
Tyr	**4.1**	0.128	**4.4**	0.306	**1.8**	0.069	**6.0**	0.317	**0.7**	0.081	**1.4**	0.040	**0.8**	0.154	**1.0**	0.927
Adenosine	**2.7**	0.237	**1.5**	0.118	**1.8**	0.006	**1.5**	0.070	**1.3**	0.442	**1.9**	0.055	**1.5**	0.055	**1.0**	0.868
XMP	**1.3**	0.690	**2.4**	0.335	**1.9**	0.018	**0.4**	0.047	**ND**		**12.9**	0.372	**0.2**	0.461	**0.2**	0.424
Dihydroorotate	**5.1**	0.010	**1.9**	0.210	**3.1**	0.179	**2.1**	0.307	**1.7**	0.312	**5.6**	0.242	**1.2**	0.176	**3.5**	0.297
Uridine	**ND**		**ND**		**37.4**	0.001	**4.1**	0.452	**ND**		**ND**		**5.0**	0.010	**2.5**	0.576
CDP	**0.3**	0.012	**0.3**	0.006	**0.5**	0.088	**0.8**	0.497	**0.5**	0.208	**1.1**	0.679	**0.5**	0.113	**0.6**	0.136
Adenylyl sulfate	**3.2**	0.242	**9.5**	0.033	**1.9**	0.141	**3.1**	0.028	**1.3**	0.472	**1.2**	0.684	**1.8**	0.066	**2.3**	0.256

In addition to decreased pantothenate, the *icc *mutant displayed other metabolite trends of a *purH *mutant including decreases in amino acid precursors (isopropylmalate, leucine biosynthesis; phosphoserine, serine biosynthesis) and increases in deoxypyrimidines (dCMP, dUMP). In total, these data suggested that in a *purH* mutant elevated cAMP was responsible for, or contributed to, these changes. Further analysis showed that the two mutants only had a minority of their statistically significant changes in common. We reasoned that in the *icc *mutant, elevated cAMP perturbed the wild-type metabolome whereas in the *purH* mutant, we observed the effects of an elevated cAMP pool in the context of a very disrupted metabolism. Our results suggested that elevated cAMP, at most, accounted for 25% of statistically significant changes observed in a *purH* mutant. This may in part reflect an inability of these approaches to adequately capture statistical significance. The *icc* mutant analysis allowed us to identify global effects of elevated cAMP pools independent of AICAR accumulation and thus defined the downstream effects of one of the major metabolic perturbations in a *purH* mutant. These results supported the potential of metabolomics analyses to modularize the pleotropic effects of genetic mutations.

### Conclusions

This study used the well-characterized *purH *mutant in *S. enterica* as a test case for metabolomics approaches to investigate bacterial physiology and to probe the global changes caused by accumulation of AICAR. AICAR is a purine biosynthetic intermediate that impacts a variety of cellular processes in diverse organisms 10,20,21,22,23,24,25,26,27,28, and is the cause of the thiamine auxotrophy in a *purH* mutant of *S. enterica*. As a proof-of-principle, metabolomics data were evaluated for a handful of metabolites that are different in *purH* and wild-type strains 10,14,15,20. With a few caveats an untargeted metabolomics approach captured known metabolic changes of a *purH *mutant, thus validating the general use of this approach.

The extent of the metabolic changes caused by removal of a single gene was unanticipated, and emphasized how dramatically AICAR levels changed the intracellular environment. Technical limitations prevented a statistically significant cataloging of all changes. To circumvent this, repeatable qualitative trends were evaluated in the context of biological knowledge and past experimental data. Despite the caveats with quantitative analyses, biological insights were gained from these metabolomics data. Firstly, an elevation of cAMP, predicted by previous genetic analyses, was detected in a *purH *mutant. The partial overlap of metabolic changes caused by a *purH *mutation and those detected in an *icc *mutant which lacks the cAMP phosphodiesterase, supported the modular description of the downstream effects that were due to cAMP accumulation rather than as a direct result of AICAR accumulation.

Secondly, mutations that suppressed the thiamine auxotrophy of a *purH *mutant caused metabolic changes that were not predictable from the framework of metabolism, but were consistent with moving the network in a *purH *mutant back toward a stable wild-type pattern. Specifically, mutations in *mtfA*, *zwf*, *and fumAC* decreased the levels of AICAR and cAMP compared to the parental *purH* mutant.

Previous studies have shown that AICAR affects diverse metabolic processes 10,20,23. Metabolomics data obtained in this study confirmed that AICAR’s physiological roles extend well beyond the PHT network in *S. enterica*. The data raised a number of issues that must be explored as we integrate biochemical genetic analysis with metabolomics approaches to understand the metabolic network in bacteria. For instance, what constitutes a physiologically relevant change in metabolite pool size? Is the 2-fold decrease in AICAR found in the suppressor mutants significant enough to account for the phenotypic switch from auxotroph to prototroph? Finally, how does one sort through the extensive data that is obtained in a global study to identify relevant information in the absence of biological context for the analyses? The latter question is particularly challenging when the much of the data are not well-replicated even with rigorous experimentation. The current study benefited from our prior knowledge of the physiological system, which allowed us to implement p-values and trend metrics such that anticipated changes were detected. To our knowledge this study is the first of its kind to probe the physiological accuracy and reproducibility of metabolomics approaches to address specific metabolic questions in bacteria. The results were mixed, in that qualitative trends were detected, but statistically significant changes were less consistent. As is the case with many high-throughput studies and well put by Dalman *et al*. with regard to transcriptomic analyses, data interpretation is “more of an art than a science, with follow up gene expression studies a must” 44. In the absence of this biological context, conclusions from qualitative metabolomics data must be made cautiously and with a clear understanding of what types of information can be gleaned from the data. Although untargeted and targeted metabolomics techniques are powerful tools, metabolite concentrations alone are not sufficient to fully describe cellular physiology; and the power of metabolomics is often better realized in combination with well-defined genetic systems and/or other data-enabled omics techniques.

### MATERIALS AND METHODS

#### Bacterial strains, media, and chemicals

Strains used in this study are derivatives of *S. enterica* serovar Typhimurium LT2 and are described in Table S1. Rich medium consisted of Difco nutrient broth (NB) (8g/L) and NaCl (5g/L). Defined medium consisted of No-carbon E medium, 1 mM MgSO_4_ 47,48,49, and trace minerals 50. Glucose (11 mM) was used as the sole carbon source. Difco BiTek agar (15 g/L) was added for solid medium. Nutrients were supplemented at the following concentrations: adenine (0.4 mM) thiamine (0.1 µM). Chemicals were purchased from Sigma-Aldrich.

#### Growth quantitation

The cells from overnight cultures (NB or defined medium) were pelleted and resuspended in an equal volume of 0.85% NaCl or for metabolomics samples, directly subcultured into the fresh defined medium. Cell density was measured as absorbance at 650 nm in a Spectronic 20 and reported as final yield at a given time.

#### Genetic techniques

Transductional crosses were performed with a high-frequency general transducing mutant of bacteriophage P22 (HT105/1, *int*-201) 51. Methods for transductional crosses, isolation of transductants from bacteriophage, and identification of bacteriophage-free transductants have been previously described 52. Mutant strains were constructed using standard genetic techniques. Insertion deletions were generated using the λ-red-mediated homologous recombination method and verified with PCR and sequence analysis 53.

#### Cyclic AMP quantification

Intracellular cyclic AMP was measured using enzyme-linked immunosorbent assay (ELISA) purchased from Cayman Chemical. Cyclic AMP concentrations were calculated using the manufacturer’s provided data analysis tools.

#### Metabolomics analysis

Three 1 mL cultures (minimal glucose adenine thiamine medium) of each strain were grown at 37˚C with shaking overnight (~12 hr). In duplicate or triplicate, a 200 µL aliquot of each overnight culture was used to inoculate 6 mL of fresh media and cultures were returned to identical growth conditions. Cell density was measured as absorbance at 650 nm (*A*_650_). Once cells reached desired growth phase one culture of each biological replicate was used for metabolite extraction. Cell densities for mid-exponential phase and early stationary phases were, *A*_650 _= 0.45-0.55 and* A*_650 _= 1.5-1.8, respectively. Early stationary phase was defined as 1 h after maximum *A*_650 _(1.5-1.8) was achieved. Late stationary phase was defined as 7 h after maximum *A*_650 _was achieved. In each experiment, all strains and biological replicates were grown and processed simultaneously.

#### Metabolite extraction

Metabolite extraction was performed generally as described 35. Cells were harvested by vacuum filtering 5 mL aliquots of each culture through Magna, nylon supported, plain 0.45 micron, 47 mm filter. Filters were transferred (cell side down) into petri dishes containing 1.3 mL of extraction solvent (40:40:20 HPLC grade methanol, acetonitrile, water with 0.1% formic acid) chilled to -20˚C. The extraction was allowed to proceed for 15 min at -20˚C. At 4˚C, and cell side of the filters were rinsed by repeatedly pipetting the extraction solvent over the face of the filters. Extracts were transferred to 1.5 mL centrifuge tubes. Filters were washed a second time with 300 µL of new extraction fluid. Filters were then discarded and extracts were added to initial extracts in 1.5 mL centrifuge tubes. Extracts were centrifuged for 5 minutes (16.1 rcf) at 4˚C and the supernatant was transferred to glass vials. The pelleted cell debris was resuspended in 50 µL of extraction solvent. The extraction was allowed to proceed for 15 min at -20˚C. Samples were centrifuged for 5 min (16.1 rcf) at 4˚C. The supernatant was added to the appropriate glass vials to combine extracts. A final extraction of the pelleted cell debris was repeated as in the previous extraction. Vials containing all of the collected supernatant were dried to completion under a stream of N_2_ at room temperature. Solid residue was resuspended in 300 µL of sterile water and transferred to 300 µL autosampler vials.

#### UHPLC-HRMS

Samples were kept at 4˚C in an Ultimate 3000 RS autosampler (Dionex, Sunnyvale, CA) until time of run. A 10 µL aliquot was injected and separated on a Synergi 2.5µ Hydro-RP 100, 100 x 2.00 mm. Untargeted metabolomics analysis was performed via a method adapted from Lu et al. 2010 36. The chromatographic gradient was from 0 to 5 min 0% B, from 5 to 13 min 20% B, from 13 to 15.5 min 55% B, from 15.5 to 19 min 95% B, and from 19 to 25 min 0% B. Solvent A was 97:3 HPLC grade water:methanol, 10 mM tributylamine, and 15 mM acetic acid. Solvent B was HPLC grade methanol. The flow rate and column compartment temperature were held constant at 200 µL/min and 25˚C, respectively. Following chromatographic elution, the samples were ionized via electrospray ionization in negative ion mode using a spray voltage of 3 kV, nitrogen sheath gas of 10 (arbitrary units), capillary temperature of 320˚C, and AGC target set to 3e6. Data was acquired using an Exactive Plus Orbitrap mass spectrometer (Thermo Scientific, Waltham, MA). The orbitrap was run in full scan mode from 85 to 800 *m/z* from 0 to 9 min and from 100 to 1000 *m/z* from 9 to 25 min using a resolution of 140,000.

#### Data analysis

Raw MS data files were converted to mzML files using ProteoWizard software 54,55. Converted spectra were processed using Metabolomic Analysis and Visualization Engine (MAVEN) 37. Metabolites were identified based on exact mass (± 5 ppm) and retention times from methods included with MAVEN 36 as well as additional in-house validated metabolites. Additional features were putatively assigned based on *m/z* and pathway analysis. For each of the “known” metabolites, extracted ion chromatograms were visualized by MAVEN and or manually integrated; Acceptable peak criteria were Guassian shaped with at least S/N and signal to blank > 3. In a given experiment, peak areas from biological triplicates were averaged and the ratios of the averages were reported as fold changes between two strains. To determine statistical significance of fold changes, an unpaired Student’s t-test (two-tailed) was applied to peak area values of biological replicates. A p-value threshold of 0.05 was used. In instances where a metabolite was only detected in 2 of 3 samples, the zero value was excluded from all calculations.

Trends were defined as instances where one or more experimental replicate illustrated statistical significance and as a whole, the majority of experiments reflected fold changes in the same direction (<0.8 or >1.2). In some instances trends are clear in one growth phase but absent or distinct in another; as these data are derived from distinct physiological scenarios they are not in conflict and both datasets may be presented for a more complete picture of the metabolome.

Partial Least Square Discriminate Analysis (PLSDA) was used as a multivariate analysis tool to provide insight into the unique metabolic fingerprint of the *purH *mutant. The 179 metabolites that were identified in the untargeted approach, were used in a pairwise fashion to generate plots and variable importance in projections (VIP) scores for the three growth phases tested. Further information can be extracted by collecting all metabolites that have VIP score greater than 1 56,57. The DiscriMiner package in R was used to perform all statistical functions, and gglplot2 was used to visualize the results 58.

## SUPPLEMENTAL MATERIAL

Click here for supplemental data file.

All supplemental data for this article are also available online at http://microbialcell.com/researcharticles/untargeted-metabolomics-confirms-and-extends-the-understanding-of-the-impact-of-aminoimidazole-carboxamide-ribotide-aicar-in-the-metabolic-network-of-salmonella-enterica/.
