# Supplementary material for: Untargeted metabolomics confirms and extends the understanding of the impact of aminoimidazole carboxamide ribotide (AICAR) in the metabolic network of Salmonella enterica
Source: Microb Cell. 2017 Nov 22;5(2):74–87. doi: 10.15698/mic2018.02.613 (PMC5798407; doi:10.15698/mic2018.02.613)
Supplement: Supplementary file 1 [file mic-05-074-s01.pdf]

SUPPLEMENTAL MATERIAL

Untargeted metabolomics confirms and extends the understanding of the impact of aminoimidazole carboxamide ribotide (AICAR) in the metabolic network of *Salmonella enterica*.

Jannell V. Bazurto[1], Stephen P. Dearth[2], Eric D. Tague[2], Shawn R. Campagna[2] and Diana M. Downs[1]*

[1]Department of Microbiology
University of Georgia
Athens, GA 30602

[2]Department of Chemistry
University of Tennessee
Knoxville, TN 37996

**TABLE S1. Bacterial strains used in this study[a]**	
Strain	Genotype[b]
DM10000	Wild-type
DM12239	Δ*purH3121*::*kan*
DM14016	Δ*purH3121*::*kan* Δ*gdhA631*::*cat*
DM14017	Δ*purH3121*::*kan* Δ*mtfA622*::*cat*
DM14018	Δ*purH3121*::*kan* *zwf21*::Tn*10*d(Tc)
DM14019	Δ*purH3121*::*kan* *zxx4150*::Tn*10*d(Tc) *fumAC1*
DM14020	Δ*purH3121*::*kan* *zxx4150*::Tn*10*d(Tc)
DM14719	Δ*icc*::*kan*

[a] All strains are *S. enterica* serovar Typhimurium LT2 variants and were constructed for this study.

[b] Tn*10*d(Tc) refers to the transposition-defective mini-Tn*10* (Tn*10* Δ16Δ17) construct [Way, 1984].

TABLE S2. Metabolome changes of *purH* mutant at different growth phases.										
	EARLY STATIONARY PHASE									
	Exp 1		Exp 2		Exp 3		Exp 4		Exp 5	
Metabolite	**Fold Change**	p-value	**Fold Change**	p-value	**Fold Change**	p-value	**Fold Change**	p-value	**Fold Change**	p-value
cAMP	**1.2**	0.281	**2.2**	0.165	**2.2**	0.035	**1.1**	0.066	**2.1**	0.022
AICARs	**407.2**	0.194	**94.4**	0.027	**164.3**	0.079	**ND**		**0.3**	0.306
AICAR	**346.6**	0.009	**367.2**	0.003	**206.0**	0.017	**124.9**	0.020	**133.0**	0.033
Pantothenate	**0.3**	0.004	**0.2**	0.002	**0.4**	0.038	**ND**		**ND**	
Pyruvate	**1.0**	0.912	**0.9**	0.831	**1.5**	0.132	**1.8**	0.205	**1.2**	0.840
Ala	**4.8**	0.404	**2.4**	0.157	**0.3**	0.345	**1.7**	0.429	**2.2**	0.095
Lactate	**2.0**	0.149	**0.8**	0.484	**ND**		**0.5**	0.610	**1.6**	0.225
2-Oxobutanoate	**2.0**	0.250	**1.2**	0.078	**1.1**	0.865	**1.1**	0.779	**1.2**	0.431
Dimethylglycine/4-Aminobutyrate	**0.3**	0.063	**0.6**	0.052	**0.4**	0.108	**1.1**	0.802	**2.1**	0.084
Ser	**0.4**	0.097	**0.7**	0.454	**0.2**	0.010	**0.4**	0.038	**1.6**	0.550
Glycerate	**1.1**	0.493	**1.1**	0.685	**1.2**	0.044	**1.9**	0.223	**1.0**	0.909
Uracil	**0.5**	0.156	**0.6**	0.550	**1.2**	0.500	**1.9**	0.037	**1.2**	0.321
Creatinine	**0.9**	0.730	**0.8**	0.327	**0.7**	0.445	**1.2**	0.674	**ND**	
Pro	**1.6**	0.430	**1.4**	0.386	**1.2**	0.605	**0.1**	0.041	**1.1**	0.879
Fumarate[1]	**1.3**	0.053	**1.3**	0.011	**1.7**	0.156	**0.7**	0.728	**4.3**	0.157
Fumarate[2]	**0.9**	0.806	**0.1**	0.014	**0.6**	0.554	**ND**		**ND**	
2-Ketoisovalerate (KIV)[1]	**1.2**	0.027	**1.2**	0.033	**1.1**	0.207	**3.0**	0.032	**1.5**	0.181
2-Ketoisovalerate (KIV)[2]	**1.2**	0.256	**1.5**	0.198	**2.3**	0.216	**1.4**	0.025	**1.3**	0.032
Guanidoacetic acid	**1.7**	0.197	**1.6**	0.449	**8.9**	0.020	**4.1**	0.093	**1.5**	0.016
Indole[1]	**2.6**	0.159	**3.2**	0.009	**5.1**	0.025	**2.8**	0.166	**3.8**	0.010
Indole[2]	**0.9**	0.957	**ND**		**1.8**	0.352	**4.9**	0.098	**2.1**	0.115
Betaine/Val	**2.5**	0.144	**2.9**	0.113	**5.0**	0.000	**3.8**	0.004	**2.3**	0.009
Methylmalonic acid	**0.9**	0.792	**0.2**	0.034	**1.1**	0.930	**0.6**	0.386	**0.9**	0.729
Homoserine/Thr	**1.3**	0.294	**1.2**	0.099	**0.9**	0.685	**1.2**	0.071	**1.2**	0.467
3-S-Methylthiopropionate	**1.9**	0.328	**1.8**	0.534	**1.8**	0.154	**1.3**	0.485	**1.8**	0.096
Nicotinamide	**0.5**	0.003	**1.9**	0.125	**1.5**	0.502	**0.8**	0.654	**1.4**	0.570
Nicotinate	**0.6**	0.085	**1.0**	0.995	**1.0**	0.944	**7.2**	0.094	**1.1**	0.929
Thymine	**2.2**	0.524	**3.4**	0.445	**2.3**	0.006	**4.2**	0.021	**1.8**	0.006
Pyroglutamic acid (5-Oxoproline)[1]	**0.9**	0.496	**0.8**	0.268	**0.7**	0.386	**0.7**	0.336	**0.5**	0.001
Pyroglutamic acid (5-Oxoproline)[2]	**1.1**	0.886	**3.9**	0.172	**1.7**	0.201	**11.0**	0.006	**4.0**	0.450
Citraconic acid	**0.9**	0.314	**1.0**	0.988	**0.9**	0.437	**1.0**	0.957	**0.9**	0.434
Hydroxyproline	**0.2**	0.095	**0.1**	0.158	**0.4**	0.044	**0.4**	0.314	**0.6**	0.196
Citraconic acid	**0.8**	0.031	**0.5**	0.062	**0.9**	0.708	**0.8**	0.205	**0.9**	0.269
Leu/Ile	**1.9**	0.351	**3.8**	0.151	**4.4**	0.002	**0.8**	0.882	**0.2**	0.266
Asn	**1.0**	0.901	**0.6**	0.031	**0.6**	0.349	**0.8**	0.160	**3.6**	0.048
Hydroxyisocaproate[1]	**0.9**	0.072	**0.9**	0.176	**0.8**	0.052	**0.9**	0.573	**0.9**	0.279
Hydroxyisocaproate[2]	**2.4**	0.404	**1.9**	0.314	**20.2**	0.020	**3.6**	0.094	**1.4**	0.469
Ornithine	**0.4**	0.212	**0.5**	0.220	**ND**		**0.1**	0.324	**3.0**	0.061
Asp	**0.8**	0.422	**0.3**	0.017	**0.4**	0.109	**0.6**	0.036	**0.5**	0.033
Malate[1]	**0.8**	0.528	**0.1**	0.045	**0.4**	0.312	**0.4**	0.000	**0.3**	0.050
Malate[2]	**0.8**	0.528	**0.1**	0.045	**0.4**	0.312	**0.1**	0.293	**0.3**	0.265
Homocysteine	**5.4**	0.200	**1.1**	0.838	**2.2**	0.121	**1.4**	0.266	**5.1**	0.085
Adenine[1]	**0.5**	0.004	**0.7**	0.149	**0.8**	0.287	**0.9**	0.202	**0.9**	0.238

Adenine[2]	**1.2**	0.806	**1.3**	0.710	**1.1**	0.601	**0.6**	0.414	**0.7**	0.129
Hypoxanthine	**0.5**	0.140	**1.1**	0.826	**0.8**	0.138	**0.7**	0.037	**0.5**	0.001
p-Hydroxybenzoate	**0.9**	0.689	**2.4**	0.341	**0.8**	0.534	**1.3**	0.356	**1.5**	0.233
Acetyl-P	**0.2**	0.000	**0.7**	0.255	**0.7**	0.318	**0.2**	0.001	**0.1**	0.036
Carbamoyl-P	**2.2**	0.296	**3.5**	0.155	**6.5**	0.042	**ND**		**ND**	
a-Ketoglutarate	**1.3**	0.399	**1.2**	0.289	**2.0**	0.023	**0.9**	0.497	**0.8**	0.224
Gln	**0.9**	0.806	**1.1**	0.755	**1.1**	0.576	**0.4**	0.090	**0.7**	0.003
Lys	**0.7**	0.129	**0.9**	0.246	**1.5**	0.375	**1.4**	0.286	**1.7**	0.258
Glu[1]	**0.9**	0.351	**0.7**	0.137	**0.8**	0.370	**0.6**	0.075	**0.5**	0.001
Glu[2]	**1.8**	0.256	**1.2**	0.798	**2.4**	0.003	**5.5**	0.044	**3.2**	0.155
2-Hydroxy-2-methylbutanedioic acid	**1.1**	0.900	**0.5**	0.254	**0.7**	0.209	**1.4**	0.153	**0.7**	0.105
Met	**0.5**	0.066	**0.5**	0.001	**0.2**	0.053	**0.7**	0.219	**1.2**	0.753
3-Methylphenylacetic acid	**0.7**	0.350	**0.9**	0.713	**0.9**	0.726	**ND**		**ND**	
Guanine	**1.4**	0.706	**2.8**	0.444	**1.9**	0.051	**ND**		**3.0**	0.020
2,3-Dihydroxybenzoic acid	**4.3**	0.402	**13.6**	0.100	**14.4**	0.008	**ND**		**ND**	
His	**1.4**	0.322	**1.4**	0.270	**0.7**	0.249	**0.9**	0.843	**0.8**	0.311
Orotate	**2.7**	0.113	**1.9**	0.171	**1.9**	0.011	**ND**		**ND**	
Dihydroorotate	**5.7**	0.210	**5.1**	0.010	**3.1**	0.179	**0.8**	0.898	**ND**	
Aminoadipate	**2.5**	0.097	**2.5**	0.114	**2.7**	0.001	**ND**		**ND**	
Carnitine	**1.0**	0.561	**1.1**	0.223	**1.0**	0.785	**ND**		**ND**	
Phenylpyruvate	**4.0**	0.008	**2.4**	0.012	**1.5**	0.101	**ND**		**ND**	
Methionine sulfoxide	**0.5**	0.085	**0.7**	0.147	**0.3**	0.305	**ND**		**ND**	
Phe	**13.4**	0.173	**6.9**	0.083	**2.9**	0.020	**ND**		**ND**	
Atrolactic acid	**1.7**	0.147	**1.5**	0.395	**3.7**	0.003	**ND**		**ND**	
Phenyllactic acid	**1.8**	0.134	**1.5**	0.379	**3.7**	0.003	**ND**		**ND**	
Phosphoenolpyruvate (PEP)	**6.5**	0.077	**0.6**	0.201	**2.5**	0.204	**ND**		**ND**	
Uric acid	**4.3**	0.421	**1.3**	0.811	**ND**		**ND**		**ND**	
Pyridoxamine	**1.0**	0.983	**1.0**	0.957	**1.2**	0.278	**1.4**	0.819	**ND**	
D-Glyceraldehdye-3-P	**3.6**	0.069	**1.7**	0.144	**4.5**	0.017	**3.8**	0.027	**2.0**	0.017
sn-Glycerol-3-P	**4.1**	0.258	**1.3**	0.183	**1.1**	0.674	**2.8**	0.245	**1.1**	0.819
Aconitate	**0.8**	0.291	**0.4**	0.033	**0.8**	0.489	**ND**		**ND**	
Shikimate	**1.3**	0.725	**1.1**	0.929	**1.7**	0.055	**ND**		**ND**	
N-Acetyl-L-ornithine	**0.0**	0.005	**0.0**	0.008	**0.1**	0.090	**1.1**	0.748	**0.8**	0.153
Arg	**2.2**	0.087	**1.3**	0.417	**0.9**	0.722	**0.1**	0.033	**0.1**	0.003
Citrulline	**0.3**	0.005	**0.7**	0.250	**0.4**	0.108	**ND**		**0.8**	0.503
Ascorbic acid	**1.2**	0.329	**1.1**	0.580	**0.9**	0.751	**3.7**	0.242	**0.4**	0.114
N-Carbamoyl-L-aspartate	**8.8**	0.152	**15.6**	0.068	**1.7**	0.064	**0.7**	0.611	**0.5**	0.418
Allantoate	**0.8**	0.864	**2.6**	0.244	**0.9**	0.841	**0.1**	0.031	**1.1**	0.881
Isopropylmalate	**0.2**	0.003	**0.1**	0.005	**0.1**	0.073	**ND**		**ND**	
Pyrophosphate	**1.5**	0.632	**1.8**	0.465	**1.9**	0.030	**ND**		**1.2**	0.882
Gluconolactone	**0.4**	0.019	**1.0**	0.913	**0.7**	0.088	**ND**		**ND**	
Glucosamine	**0.5**	0.177	**0.9**	0.772	**0.8**	0.318	**ND**		**ND**	
Hydroxyphenylpyruvate	**0.7**	0.235	**1.1**	0.520	**1.6**	0.028	**ND**		**ND**	
Myo-inositol	**0.5**	0.018	**0.7**	0.000	**0.6**	0.159	**ND**		**6.6**	0.155
Tyr	**9.6**	0.198	**4.1**	0.128	**1.8**	0.069	**ND**		**0.4**	0.392
4-Pyridoxic acid	**0.9**	0.555	**1.0**	0.728	**1.1**	0.457	**ND**		**ND**	
Phosphoserine	**0.1**	0.077	**0.0**	0.030	**0.1**	0.023	**ND**		**ND**	
Phosphoglycerate	**3.2**	0.059	**0.7**	0.389	**1.7**	0.220	**0.4**	0.644	**ND**	
N-Acetyl-glutamine	**0.6**	0.240	**0.7**	0.351	**0.9**	0.541	**ND**		**1.2**	0.923
Acetyllysine	**0.1**	0.038	**0.3**	0.005	**0.2**	0.003	**0.1**	0.012	**0.8**	0.556
Kynurenic acid	**4.0**	0.078	**1.5**	0.489	**3.5**	0.016	**ND**		**ND**	
N-Acetyl-glutamate[1]	**0.6**	0.189	**1.1**	0.878	**1.6**	0.020	**ND**		**ND**	

N-Acetyl-glutamate[2]	**0.9**	0.900	**1.0**	0.789	**1.9**	0.021	**ND**		**ND**	
Citrate	**0.4**	0.076	**0.2**	0.070	**0.2**	0.153	**ND**		**0.6**	0.088
2-Dehydro-D-gluconate	**1.8**	0.361	**2.4**	0.391	**1.5**	0.010	**ND**		**ND**	
D-Gluconate	**0.1**	0.399	**2.0**	0.486	**5.5**	0.287	**0.7**	0.326	**0.7**	0.227
Trp	**3.0**	0.244	**2.2**	0.132	**1.0**	0.875	**ND**		**ND**	
Acetylcarnitine	**ND**		**ND**		**ND**		**0.6**	0.225	**0.8**	0.502
Xanthurenic acid	**0.8**	0.515	**0.9**	0.882	**1.7**	0.511	**1.9**	0.152	**1.6**	0.143
Lipoate	**0.3**	0.180	**0.4**	0.033	**0.7**	0.498	**ND**		**ND**	
Kynurenine	**1.2**	0.572	**0.8**	0.217	**1.4**	0.355	**1.1**	0.765	**0.9**	0.467
D-Glucarate[1]	**2.6**	0.402	**1.1**	0.752	**ND**		**1.3**	0.471	**1.0**	0.967
D-Glucarate[2]	**0.8**	0.207	**0.7**	0.315	**1.0**	0.905	**ND**		**ND**	
Deoxyribose-P	**2.3**	0.038	**0.6**	0.070	**1.6**	0.569	**5.3**	0.038	**0.6**	0.017
Cystathionine	**0.4**	0.175	**1.0**	0.949	**ND**		**ND**		**ND**	
Prephenate	**5.0**	0.148	**2.6**	0.355	**2.3**	0.006	**ND**		**ND**	
Deoxyuridine	**0.2**	0.111	**1.2**	0.529	**1.2**	0.209	**0.9**	0.662	**3.3**	0.104
Ribose-P	**1.4**	0.584	**5.0**	0.124	**36.6**	0.060	**ND**		**ND**	
Thymidine	**0.1**	0.299	**0.2**	0.345	**ND**		**1.5**	0.375	**0.9**	0.737
Cytidine	**1.5**	0.451	**9.7**	0.029	**1.6**	0.415	**ND**		**ND**	
Uridine	**3.0**	0.204	**12.1**	0.027	**37.4**	0.001	**ND**		**1.2**	0.202
Deoxyinosine	**0.3**	0.145	**0.6**	0.416	**3.5**	0.125	**37.8**	0.111	**2.0**	0.067
Shikimate-3-P	**1.2**	0.812	**0.8**	0.840	**1.8**	0.081	**2.8**	0.212	**ND**	
D-Gluconolactone-6-P	**0.9**	0.665	**1.2**	0.362	**0.9**	0.537	**ND**		**ND**	
Glycerophosphocholine	**0.8**	0.219	**1.0**	0.818	**1.2**	0.461	**10.9**	0.003	**4.7**	0.017
D-Glucosamine-1-P	**2.1**	0.228	**2.8**	0.220	**2.8**	0.003	**ND**		**ND**	
Glucose-1-P	**1.2**	0.592	**3.0**	0.192	**0.7**	0.105	**0.2**	0.055	**1.0**	0.963
Thiamine	**0.4**	0.357	**0.2**	0.051	**ND**		**ND**		**0.0**	0.131
2,3-Diphosphoglyceric acid[1]	**6.8**	0.068	**1.8**	0.028	**2.5**	0.373	**ND**		**ND**	
2,3-Diphosphoglyceric acid[2]	**0.9**	0.849	**1.1**	0.707	**2.2**	0.137	**0.1**	0.004	**0.5**	0.038
S-Ribosylhomocysteine (SRH)	**1.7**	0.045	**1.2**	0.306	**3.1**	0.001	**1.7**	0.027	**1.4**	0.006
Adenosine	**1.4**	0.568	**2.7**	0.237	**1.8**	0.006	**1.4**	0.093	**1.2**	0.098
Inosine	**2.5**	0.002	**1.1**	0.767	**2.9**	0.114	**1.2**	0.706	**1.7**	0.053
6-Phosphogluconate	**0.4**	0.524	**3.6**	0.301	**0.9**	0.746	**ND**		**ND**	
1-Methyladenosine[1]	**1.3**	0.102	**4.8**	0.212	**ND**		**1.6**	0.581	**0.9**	0.935
1-Methyladenosine[2]	**1.2**	0.333	**1.1**	0.484	**1.2**	0.401	**1.9**	0.015	**1.3**	0.418
Guanosine	**ND**		**0.3**	0.443	**ND**		**6.3**	0.021	**3.0**	0.506
Xanthosine	**4.8**	0.169	**4.9**	0.184	**2.6**	0.003	**4.5**	0.007	**4.1**	0.016
Argininosuccinate	**0.9**	0.790	**0.3**	0.002	**0.6**	0.337	**0.6**	0.299	**0.7**	0.328
N-Acetyl-glucosamine	**0.8**	0.284	**0.7**	0.114	**0.9**	0.705	**0.4**	0.095	**0.4**	0.025
Glutathione	**0.9**	0.723	**0.9**	0.702	**0.8**	0.608	**ND**		**ND**	
dUMP	**14.3**	0.000	**22.2**	0.048	**9.8**	0.013	**29.3**	0.058	**18.2**	0.059
Octoluse 8,1-P	**0.6**	0.485	**0.5**	0.370	**1.9**	0.217	**ND**		**0.7**	0.838
dTMP	**0.8**	0.371	**0.9**	0.766	**1.3**	0.161	**0.8**	0.057	**1.3**	0.373
CMP	**0.7**	0.327	**0.4**	0.061	**0.6**	0.015	**11.6**	0.057	**2.1**	0.021
UMP[1]	**1.2**	0.382	**1.0**	0.694	**0.9**	0.773	**0.6**	0.009	**0.7**	0.169
UMP[2]	**1.1**	0.730	**0.8**	0.160	**0.8**	0.355	**1.8**	0.048	**0.5**	0.142
dAMP	**1.5**	0.118	**0.9**	0.691	**2.6**	0.616	**ND**		**ND**	
Nicotinamide ribotide	**1.4**	0.410	**2.8**	0.100	**2.1**	0.002	**ND**		**ND**	
Cellobiose	**0.6**	0.102	**1.2**	0.045	**0.5**	0.047	**1.3**	0.047	**0.9**	0.248
Trehalose/Sucrose	**0.6**	0.102	**1.2**	0.045	**0.5**	0.047	**1.3**	0.047	**0.9**	0.248
AMP	**1.4**	0.044	**1.2**	0.275	**0.9**	0.486	**1.1**	0.692	**1.1**	0.781
IMP[1]	**1.2**	0.868	**1.9**	0.408	**0.4**	0.203	**ND**		**0.7**	0.718
IMP[2]	**ND**		**0.4**	0.263	**ND**		**0.3**	0.194	**0.5**	0.418

GMP	**1.7**	0.354	**0.7**	0.048	**0.6**	0.153	**1.3**	0.569	**0.9**	0.893
XMP	**3.8**	0.090	**1.3**	0.690	**1.9**	0.018	**1.2**	0.638	**0.3**	0.069
Riboflavin	**3.3**	0.390	**2.8**	0.402	**1.5**	0.220	**0.8**	0.002	**1.1**	0.175
S-Adenosylhomocysteine (SAH)	**1.0**	0.909	**1.2**	0.913	**6.3**	0.000	**1.8**	0.157	**1.0**	0.967
dCDP	**7.8**	0.455	**0.3**	0.198	**0.8**	0.273	**1.2**	0.531	**0.8**	0.357
dTDP	**4.4**	0.347	**1.4**	0.537	**0.9**	0.723	**1.2**	0.239	**1.0**	0.969
CDP	**1.1**	0.926	**0.3**	0.012	**0.5**	0.088	**1.9**	0.037	**0.4**	0.045
UDP	**5.1**	0.356	**1.6**	0.556	**1.3**	0.145	**1.6**	0.108	**0.8**	0.212
Trehalose-6-P	**0.3**	0.162	**0.8**	0.597	**ND**		**1.3**	0.103	**2.4**	0.185
Adenylyl sulfate	**1.8**	0.352	**3.2**	0.242	**1.9**	0.141	**0.8**	0.629	**2.8**	0.567
ADP	**4.1**	0.309	**2.0**	0.156	**1.2**	0.045	**1.5**	0.105	**1.7**	0.085
GDP	**6.3**	0.219	**0.9**	0.914	**0.5**	0.050	**0.8**	0.386	**0.8**	0.578
Deoxycholic acid	**0.4**	0.524	**1.0**	0.619	**1.1**	0.110	**0.7**	0.036	**0.9**	0.085
FMN	**1.5**	0.261	**1.0**	0.973	**1.1**	0.728	**0.8**	0.457	**0.9**	0.454
Cholesteryl sulfate	**0.8**	0.112	**1.0**	0.858	**0.8**	0.008	**0.5**	0.100	**0.9**	0.069
dCTP	**1.2**	0.596	**1.0**	0.942	**0.6**	0.052	**0.8**	0.325	**1.3**	0.265
dTTP	**1.1**	0.565	**0.9**	0.234	**0.9**	0.565	**0.9**	0.415	**1.0**	0.764
CTP	**1.0**	0.993	**0.5**	0.203	**0.4**	0.028	**2.9**	0.050	**0.7**	0.139
UTP	**2.3**	0.291	**2.1**	0.502	**0.5**	0.127	**2.7**	0.061	**1.6**	0.156
dATP	**0.9**	0.877	**1.5**	0.518	**0.9**	0.461	**1.2**	0.266	**2.6**	0.040
ATP	**1.6**	0.178	**1.4**	0.607	**0.5**	0.050	**1.5**	0.102	**3.1**	0.063
GTP	**1.1**	0.911	**1.6**	0.613	**0.5**	0.012	**0.9**	0.534	**1.2**	0.455
UDP-D-glucose	**4.9**	0.068	**13.2**	0.086	**2.0**	0.189	**0.8**	0.631	**0.8**	0.620
UDP-D-glucuronate	**5.7**	0.307	**ND**		**2.0**	0.001	**1.4**	0.175	**1.7**	0.035
UDP-N-acetyl-glucosamine	**3.0**	0.298	**5.0**	0.305	**1.3**	0.354	**1.0**	0.994	**1.1**	0.681
Glutathione disulfide	**1.3**	0.722	**0.9**	0.554	**1.5**	0.082	**0.7**	0.609	**0.5**	0.099
NAD+	**1.1**	0.200	**1.3**	0.110	**1.0**	0.982	**ND**		**ND**	
NADH	**0.3**	0.558	**0.8**	0.887	**1.6**	0.060	**3.6**	0.010	**0.3**	0.074
NADP+	**1.9**	0.265	**1.6**	0.342	**1.5**	0.062	**1.3**	0.064	**1.9**	0.103
NADPH	**ND**		**0.6**	0.551	**0.9**	0.701	**3.1**	0.026	**1.5**	0.117
FAD	**2.4**	0.152	**2.0**	0.327	**2.4**	0.017	**1.5**	0.001	**1.4**	0.188
Acetyl-CoA	**1.3**	0.718	**0.5**	0.058	**0.5**	0.113	**1.3**	0.367	**0.8**	0.111
	LATE STATIONARY PHASE									
	Exp 1		Exp 2		Exp 3		Exp 4		Exp 5	
cAMP	**2.4**	0.011	**1.3**	0.105	**1.6**	0.005	**2.7**	0.092	**1.7**	0.071
AICARs	**2.8**	0.011	**3.4**	0.395	**7.4**	0.023	**1.1**	0.946	**0.5**	0.423
AICAR	**16.8**	0.116	**34.1**	0.032	**32.7**	0.007	**82.0**	0.053	**47.9**	0.055
Pantothenate	**0.8**	0.607	**0.2**	0.000	**0.2**	0.000	**ND**		**ND**	
Pyruvate	**0.7**	0.321	**1.3**	0.263	**0.9**	0.672	**2.4**	0.300	**0.8**	0.510
Ala	**1.0**	0.938	**1.1**	0.909	**0.4**	0.440	**1.9**	0.557	**0.5**	0.158
Lactate	**0.3**	0.380	**0.5**	0.620	**1.5**	0.262	**1.5**	0.649	**0.9**	0.627
2-Oxobutanoate	**1.8**	0.114	**0.7**	0.193	**0.8**	0.623	**3.8**	0.381	**0.9**	0.599
Dimethylglycine/4-Aminobutyrate	**0.5**	0.355	**0.5**	0.330	**0.5**	0.011	**0.1**	0.029	**0.0**	0.000
Ser	**0.8**	0.558	**1.4**	0.017	**0.4**	0.308	**0.9**	0.748	**3.2**	0.250
Glycerate	**0.5**	0.020	**1.0**	0.986	**1.1**	0.726	**0.1**	0.000	**0.4**	0.010
Uracil	**1.1**	0.698	**0.9**	0.296	**1.3**	0.009	**2.0**	0.243	**1.2**	0.213
Creatinine	**1.3**	0.561	**0.5**	0.202	**1.3**	0.588	**14.6**	0.304	**ND**	
Pro	**0.8**	0.703	**0.8**	0.615	**1.3**	0.493	**1.2**	0.831	**3.6**	0.209
Fumarate[1]	**1.9**	0.026	**0.9**	0.644	**1.1**	0.290	**1.0**	0.987	**3.3**	0.482
Fumarate[2]	**0.2**	0.157	**0.6**	0.078	**0.3**	0.332	**0.5**	0.491	**ND**	
2-Ketoisovalerate (KIV)[1]	**0.7**	0.648	**1.5**	0.259	**1.1**	0.376	**1.2**	0.554	**1.5**	0.319
2-Ketoisovalerate (KIV)[2]	**1.2**	0.347	**1.6**	0.032	**1.0**	0.601	**4.1**	0.050	**1.7**	0.288
Guanidoacetic acid	**2.2**	0.060	**2.2**	0.233	**4.7**	0.142	**5.2**	0.206	**2.7**	0.084

Indole[1]	**2.3**	0.033	**4.1**	0.038	**7.1**	0.055	**5.4**	0.185	**2.9**	0.155
Indole[2]	**1.2**	0.687	**0.9**	0.942	**2.5**	0.063	**3.9**	0.195	**2.1**	0.035
Betaine/Val	**1.9**	0.012	**2.3**	0.001	**3.6**	0.010	**6.1**	0.069	**2.9**	0.000
Methylmalonic acid	**0.9**	0.116	**0.9**	0.051	**1.1**	0.715	**1.7**	0.264	**1.4**	0.528
Homoserine/Thr	**0.7**	0.039	**0.9**	0.536	**0.9**	0.776	**1.4**	0.486	**1.1**	0.857
3-S-Methylthiopropionate	**0.7**	0.541	**1.2**	0.597	**1.6**	0.074	**1.5**	0.385	**1.1**	0.562
Nicotinamide	**0.8**	0.586	**1.4**	0.320	**1.7**	0.297	**1.3**	0.506	**1.4**	0.538
Nicotinate	**1.9**	0.256	**1.1**	0.742	**1.1**	0.723	**1.3**	0.697	**3.1**	0.268
Thymine	**0.7**	0.361	**0.8**	0.433	**1.2**	0.220	**0.9**	0.604	**1.1**	0.568
Pyroglutamic acid (5-Oxoproline)[1]	**0.2**	0.079	**0.4**	0.012	**0.4**	0.007	**0.8**	0.692	**0.8**	0.408
Pyroglutamic acid (5-Oxoproline)[2]	**0.7**	0.462	**0.7**	0.221	**0.7**	0.498	**1.6**	0.369	**8.4**	0.215
Citraconic acid	**0.9**	0.251	**1.0**	0.928	**0.9**	0.035	**0.6**	0.193	**1.0**	0.901
Hydroxyproline	**0.2**	0.279	**0.5**	0.012	**0.4**	0.003	**0.8**	0.815	**2.5**	0.178
Citraconic acid	**0.8**	0.494	**0.4**	0.064	**0.6**	0.010	**1.4**	0.425	**0.9**	0.548
Leu/Ile	**1.1**	0.661	**1.1**	0.528	**2.9**	0.010	**3437.8**	0.500	**ND**	
Asn	**2.0**	0.030	**1.1**	0.644	**0.8**	0.435	**1.4**	0.202	**1.9**	0.281
Hydroxyisocaproate[1]	**0.8**	0.108	**0.9**	0.603	**0.8**	0.037	**5.9**	0.027	**1.5**	0.290
Hydroxyisocaproate[2]	**1.0**	0.818	**1.3**	0.089	**6.2**	0.002	**5.5**	0.171	**8.9**	0.139
Ornithine	**0.4**	0.269	**0.7**	0.272	**ND**		**0.2**	0.075	**0.8**	0.646
Asp	**0.3**	0.021	**0.3**	0.001	**0.4**	0.013	**0.7**	0.022	**1.1**	0.810
Malate[1]	**0.1**	0.105	**0.3**	0.186	**0.2**	0.178	**1.6**	0.449	**0.3**	0.082
Malate[2]	**0.1**	0.105	**0.3**	0.186	**0.1**	0.169	**1.8**	0.507	**0.0**	0.327
Homocysteine	**ND**		**1.5**	0.153	**ND**		**0.7**	0.455	**0.2**	0.011
Adenine[1]	**1.1**	0.704	**1.5**	0.038	**1.2**	0.588	**1.7**	0.260	**0.9**	0.019
Adenine[2]	**1.1**	0.722	**1.7**	0.027	**1.9**	0.003	**1.5**	0.331	**0.9**	0.451
Hypoxanthine	**0.5**	0.046	**0.5**	0.081	**0.7**	0.001	**0.9**	0.586	**0.6**	0.028
p-Hydroxybenzoate	**0.8**	0.385	**0.6**	0.414	**0.8**	0.027	**2.9**	0.025	**1.0**	0.951
Acetyl-P	**0.1**	0.220	**0.1**	0.085	**0.1**	0.032	**2.4**	0.272	**0.0**	0.020
Carbamoyl-P	**0.1**	0.055	**0.5**	0.330	**0.4**	0.091	**ND**		**ND**	
a-Ketoglutarate	**1.1**	0.923	**0.4**	0.056	**1.8**	0.125	**0.6**	0.406	**0.3**	0.055
Gln	**0.4**	0.015	**0.9**	0.591	**0.7**	0.511	**2.2**	0.000	**0.4**	0.004
Lys	**0.5**	0.254	**0.4**	0.004	**0.8**	0.476	**0.8**	0.599	**2.8**	0.101
Glu[1]	**0.3**	0.054	**0.5**	0.011	**0.5**	0.006	**1.3**	0.157	**0.8**	0.330
Glu[2]	**0.6**	0.009	**1.2**	0.785	**0.9**	0.518	**1.3**	0.557	**1.9**	0.212
2-Hydroxy-2-methylbutanedioic acid	**0.5**	0.219	**0.2**	0.060	**0.6**	0.015	**1.1**	0.841	**0.6**	0.074
Met	**0.9**	0.514	**1.3**	0.609	**0.6**	0.052	**0.2**	0.161	**2.2**	0.290
3-Methylphenylacetic acid	**1.2**	0.276	**1.4**	0.180	**1.2**	0.599	**ND**		**ND**	
Guanine	**1.0**	0.929	**0.8**	0.091	**1.5**	0.001	**0.6**	0.235	**1.4**	0.729
2,3-Dihydroxybenzoic acid	**1.0**	0.974	**1.6**	0.560	**3.7**	0.441	**ND**		**0.3**	0.131
His	**0.2**	0.019	**0.6**	0.247	**0.6**	0.583	**3.1**	0.434	**0.8**	0.203
Orotate	**3.2**	0.339	**1.4**	0.615	**0.5**	0.573	**4.6**	0.519	**ND**	
Dihydroorotate	**0.8**	0.666	**1.7**	0.312	**1.2**	0.176	**ND**		**ND**	
Aminoadipate	**1.1**	0.459	**1.8**	0.005	**2.0**	0.021	**ND**		**ND**	
Carnitine	**0.8**	0.112	**1.0**	0.370	**0.9**	0.548	**ND**		**ND**	
Phenylpyruvate	**0.2**	0.309	**1.3**	0.149	**1.0**	0.784	**ND**		**ND**	
Methionine sulfoxide	**0.9**	0.843	**5.8**	0.059	**1.2**	0.802	**ND**		**ND**	
Phe	**0.4**	0.046	**0.7**	0.018	**0.8**	0.276	**ND**		**5.3**	0.406
Atrolactic acid	**1.0**	0.909	**1.5**	0.205	**1.4**	0.066	**ND**		**ND**	
Phenyllactic acid	**1.0**	0.909	**1.5**	0.205	**1.4**	0.066	**ND**		**ND**	
Phosphoenolpyruvate (PEP)	**0.2**	0.172	**0.7**	0.320	**0.4**	0.010	**ND**		**ND**	
Uric acid	**4.1**	0.422	**0.2**	0.426	**ND**		**ND**		**ND**	

Pyridoxamine	**1.2**	0.557	**1.0**	0.957	**0.9**	0.826	**ND**		**ND**	
D-Glyceraldehdye-3-P	**0.1**	0.161	**2.3**	0.031	**1.7**	0.129	**1.7**	0.572	**0.8**	0.355
sn-Glycerol-3-P	**0.4**	0.113	**1.9**	0.045	**0.4**	0.215	**0.5**	0.635	**1.1**	0.891
Aconitate	**0.8**	0.432	**0.4**	0.021	**0.6**	0.050	**ND**		**ND**	
Shikimate	**1.0**	0.974	**1.0**	0.843	**1.5**	0.015	**ND**		**0.3**	0.098
N-Acetyl-L-ornithine	**0.1**	0.011	**0.2**	0.168	**0.1**	0.103	**2.7**	0.440	**0.9**	0.239
Arg	**0.4**	0.021	**0.4**	0.011	**1.0**	0.906	**0.1**	0.011	**1.2**	0.795
Citrulline	**1.1**	0.854	**1.1**	0.638	**1.9**	0.036	**0.3**	0.175	**1.9**	0.104
Ascorbic acid	**0.8**	0.508	**0.7**	0.448	**0.9**	0.505	**0.1**	0.077	**0.0**	0.006
N-Carbamoyl-L-aspartate	**0.5**	0.283	**0.7**	0.232	**0.7**	0.019	**0.8**	0.711	**1.3**	0.773
Allantoate	**0.4**	0.403	**0.8**	0.872	**1.0**	0.935	**2.0**	0.398	**1.0**	0.937
Isopropylmalate	**3.5**	0.319	**4.1**	0.002	**0.6**	0.068	**ND**		**ND**	
Pyrophosphate	**0.9**	0.749	**1.0**	0.858	**1.0**	0.618	**ND**		**ND**	
Gluconolactone	**2.5**	0.166	**1.4**	0.242	**1.0**	0.832	**ND**		**ND**	
Glucosamine	**0.5**	0.116	**0.6**	0.359	**0.9**	0.582	**ND**		**ND**	
Hydroxyphenylpyruvate	**0.9**	0.763	**1.0**	0.804	**1.2**	0.258	**ND**		**ND**	
Myo-inositol	**0.8**	0.131	**0.8**	0.380	**0.8**	0.105	**1.7**	0.258	**3.5**	0.306
Tyr	**0.4**	0.011	**0.7**	0.081	**0.8**	0.154	**ND**		**1.2**	0.782
4-Pyridoxic acid	**0.5**	0.327	**0.9**	0.181	**0.4**	0.030	**ND**		**ND**	
Phosphoserine	**0.4**	0.010	**5.1**	0.048	**1.1**	0.756	**ND**		**ND**	
Phosphoglycerate	**0.1**	0.152	**1.0**	0.953	**0.5**	0.011	**ND**		**ND**	
N-Acetyl-glutamine	**0.1**	0.040	**0.2**	0.016	**0.4**	0.018	**ND**		**ND**	
Acetyllysine	**0.2**	0.007	**0.2**	0.016	**0.3**	0.186	**1.1**	0.812	**1.0**	0.925
Kynurenic acid	**1.6**	0.336	**0.6**	0.350	**2.3**	0.030	**ND**		**ND**	
N-Acetyl-glutamate[1]	**1.1**	0.802	**1.0**	0.970	**1.0**	0.450	**ND**		**ND**	
N-Acetyl-glutamate[2]	**0.2**	0.004	**0.9**	0.679	**0.7**	0.203	**ND**		**ND**	
Citrate	**0.6**	0.354	**0.4**	0.173	**0.3**	0.001	**ND**		**ND**	
2-Dehydro-D-gluconate	**1.0**	0.989	**1.4**	0.720	**1.2**	0.281	**ND**		**ND**	
D-Gluconate	**0.7**	0.304	**0.8**	0.558	**0.8**	0.851	**0.8**	0.401	**1.2**	0.726
Trp	**0.4**	0.033	**0.5**	0.190	**0.9**	0.505	**ND**		**ND**	
Acetylcarnitine	**2.3**	0.691	**ND**		**ND**		**1.2**	0.718	**0.5**	0.087
Xanthurenic acid	**0.6**	0.075	**0.7**	0.503	**1.2**	0.482	**1.7**	0.446	**1.4**	0.439
Lipoate	**66.9**	0.127	**2.8**	0.276	**1.7**	0.445	**ND**		**0.2**	0.069
Kynurenine	**0.8**	0.633	**0.6**	0.229	**1.0**	0.996	**4.8**	0.387	**0.9**	0.175
D-Glucarate[1]	**7.9**	0.318	**ND**		**ND**		**1.0**	0.891	**0.8**	0.190
D-Glucarate[2]	**0.9**	0.367	**0.7**	0.375	**0.8**	0.137	**1.1**	0.963	**ND**	
Deoxyribose-P	**1.0**	0.938	**1.1**	0.549	**1.0**	0.953	**0.4**	0.438	**1.8**	0.271
Cystathionine	**0.9**	0.763	**3.6**	0.203	**ND**		**ND**		**ND**	
Prephenate	**0.9**	0.961	**0.6**	0.584	**1.3**	0.627	**ND**		**ND**	
Deoxyuridine	**1.3**	0.546	**0.9**	0.456	**1.0**	0.869	**1.9**	0.436	**1.8**	0.238
Ribose-P	**0.1**	0.156	**2.1**	0.078	**1.5**	0.103	**ND**		**ND**	
Thymidine	**0.2**	0.034	**0.3**	0.118	**ND**		**0.2**	0.078	**0.2**	0.034
Cytidine	**5.9**	0.054	**10.9**	0.070	**24.0**	0.373	**ND**		**1.1**	0.946
Uridine	**0.6**	0.363	**0.6**	0.376	**5.0**	0.010	**1.7**	0.736	**0.5**	0.578
Deoxyinosine	**13.4**	0.240	**1.5**	0.139	**0.4**	0.287	**20.7**	0.136	**12.6**	0.147
Shikimate-3-P	**3.2**	0.150	**13.0**	0.023	**2.0**	0.004	**1.3**	0.721	**0.7**	0.238
D-Gluconolactone-6-P	**0.8**	0.461	**1.1**	0.952	**1.1**	0.829	**ND**		**ND**	
Glycerophosphocholine	**0.8**	0.155	**0.8**	0.253	**0.7**	0.330	**16.2**	0.242	**9.6**	0.013
D-Glucosamine-1-P	**0.9**	0.711	**1.6**	0.058	**2.2**	0.053	**ND**		**ND**	
Glucose-1-P	**0.1**	0.150	**5.0**	0.127	**0.7**	0.239	**1.3**	0.592	**0.6**	0.414
Thiamine	**0.1**	0.049	**ND**		**ND**		**ND**		**ND**	
2,3-Diphosphoglyceric acid[1]	**2.9**	0.065	**0.8**	0.862	**0.5**	0.670	**ND**		**ND**	
2,3-Diphosphoglyceric acid[2]	**0.6**	0.275	**ND**		**0.9**	0.706	**2.3**	0.178	**1.3**	0.701

S-Ribosylhomocysteine (SRH)	**1.8**	0.052	**1.6**	0.001	**3.7**	0.010	**1.3**	0.594	**1.0**	0.896
Adenosine	**1.1**	0.755	**1.3**	0.442	**1.5**	0.055	**0.7**	0.164	**0.8**	0.273
Inosine	**1.7**	0.340	**1.2**	0.653	**6.4**	0.071	**0.8**	0.456	**1.0**	0.955
6-Phosphogluconate	**0.5**	0.458	**1.5**	0.546	**0.7**	0.219	**ND**		**1.1**	0.622
1-Methyladenosine[1]	**1.6**	0.945	**ND**		**0.4**	0.464	**2.4**	0.442	**4.8**	0.254
1-Methyladenosine[2]	**1.8**	0.349	**1.4**	0.115	**0.8**	0.441	**0.7**	0.211	**1.2**	0.521
Guanosine	**0.9**	0.941	**0.9**	0.785	**0.5**	0.030	**1.6**	0.507	**6.1**	0.213
Xanthosine	**2.7**	0.083	**4.2**	0.003	**3.3**	0.018	**5.3**	0.058	**2.4**	0.025
Argininosuccinate	**0.2**	0.050	**0.4**	0.009	**0.6**	0.274	**1.4**	0.039	**0.7**	0.435
N-Acetyl-glucosamine	**0.6**	0.211	**2.0**	0.026	**0.7**	0.220	**1.8**	0.353	**0.8**	0.350
Glutathione	**1.5**	0.498	**0.7**	0.738	**0.8**	0.455	**ND**		**0.1**	0.496
dUMP	**2.7**	0.155	**0.9**	0.576	**1.1**	0.830	**7.5**	0.236	**3.5**	0.013
Octoluse 8,1-P	**1.1**	0.783	**1.6**	0.086	**2.0**	0.040	**ND**		**ND**	
dTMP	**0.9**	0.466	**0.6**	0.361	**0.8**	0.196	**1.1**	0.610	**0.7**	0.034
CMP	**1.9**	0.033	**0.5**	0.016	**0.6**	0.063	**4.2**	0.052	**2.2**	0.050
UMP[1]	**0.9**	0.560	**0.6**	0.067	**0.6**	0.030	**0.7**	0.726	**0.2**	0.108
UMP[2]	**0.8**	0.556	**0.6**	0.016	**0.7**	0.306	**0.7**	0.201	**0.3**	0.020
dAMP	**0.3**	0.161	**0.5**	0.265	**0.9**	0.746	**ND**		**ND**	
Nicotinamide ribotide	**1.8**	0.033	**2.0**	0.097	**1.3**	0.671	**ND**		**ND**	
Cellobiose	**1.0**	0.890	**0.7**	0.017	**0.6**	0.173	**3.6**	0.005	**1.1**	0.068
Trehalose/Sucrose	**1.0**	0.890	**0.7**	0.017	**0.6**	0.173	**3.6**	0.005	**1.1**	0.068
AMP	**1.5**	0.356	**0.6**	0.203	**0.9**	0.623	**0.8**	0.258	**0.4**	0.013
IMP[1]	**1.4**	0.223	**0.8**	0.875	**ND**		**ND**		**2.5**	0.436
IMP[2]	**ND**		**ND**		**ND**		**1.9**	0.243	**0.5**	0.085
GMP	**1.7**	0.400	**0.4**	0.339	**0.4**	0.230	**0.8**	0.324	**0.8**	0.427
XMP	**ND**		**0.4**	0.424	**0.2**	0.461	**4.6**	0.106	**1.6**	0.350
Riboflavin	**0.2**	0.153	**0.4**	0.323	**0.6**	0.025	**0.9**	0.550	**1.2**	0.035
S-Adenosylhomocysteine (SAH)	**0.7**	0.139	**0.2**	0.052	**0.4**	0.080	**2.6**	0.596	**1.3**	0.718
dCDP	**1.7**	0.389	**0.8**	0.476	**0.8**	0.062	**0.9**	0.610	**0.5**	0.009
dTDP	**1.5**	0.131	**0.7**	0.588	**0.7**	0.072	**1.1**	0.763	**1.2**	0.092
CDP	**1.3**	0.574	**0.5**	0.208	**0.5**	0.113	**1.9**	0.133	**0.7**	0.033
UDP	**0.8**	0.627	**0.7**	0.404	**0.6**	0.054	**1.8**	0.237	**0.8**	0.180
Trehalose-6-P	**5.6**	0.039	**1.5**	0.281	**3.4**	0.066	**11.4**	0.072	**3.0**	0.098
Adenylyl sulfate	**1.2**	0.801	**1.3**	0.472	**1.8**	0.066	**7.6**	0.360	**1.4**	0.369
ADP	**1.0**	0.757	**0.9**	0.536	**0.8**	0.088	**1.4**	0.400	**0.9**	0.164
GDP	**0.6**	0.479	**1.4**	0.048	**0.5**	0.042	**1.4**	0.408	**0.7**	0.006
Deoxycholic acid	**1.0**	0.615	**1.0**	0.784	**1.1**	0.635	**3.2**	0.222	**1.2**	0.545
FMN	**0.8**	0.183	**0.7**	0.061	**0.7**	0.357	**1.9**	0.272	**0.7**	0.100
Cholesteryl sulfate	**1.0**	0.599	**1.0**	0.961	**1.0**	0.811	**4.7**	0.343	**0.9**	0.558
dCTP	**0.4**	0.087	**2.2**	0.146	**1.0**	0.863	**1.4**	0.477	**0.8**	0.209
dTTP	**0.8**	0.252	**1.2**	0.031	**1.1**	0.852	**0.9**	0.726	**0.8**	0.018
CTP	**0.6**	0.290	**1.5**	0.025	**0.7**	0.397	**2.5**	0.161	**0.7**	0.078
UTP	**0.8**	0.753	**2.0**	0.176	**1.0**	0.972	**2.3**	0.225	**0.8**	0.164
dATP	**0.7**	0.464	**1.5**	0.116	**0.9**	0.323	**2.1**	0.212	**1.3**	0.215
ATP	**0.4**	0.133	**1.6**	0.333	**1.0**	0.825	**1.6**	0.344	**1.0**	0.883
GTP	**0.6**	0.548	**4.4**	0.185	**1.0**	0.906	**1.9**	0.342	**0.7**	0.105
UDP-D-glucose	**0.0**	0.153	**3.0**	0.050	**0.5**	0.428	**4.1**	0.088	**0.8**	0.601
UDP-D-glucuronate	**0.1**	0.054	**ND**		**0.2**	0.001	**3.5**	0.087	**0.5**	0.008
UDP-N-acetyl-glucosamine	**0.4**	0.145	**2.4**	0.031	**0.9**	0.703	**1.2**	0.593	**0.6**	0.096
Glutathione disulfide	**3.6**	0.063	**2.0**	0.200	**2.6**	0.047	**2.1**	0.201	**0.4**	0.400
NAD+	**1.1**	0.504	**0.9**	0.248	**1.0**	0.948	**ND**		**ND**	
NADH	**0.6**	0.551	**0.9**	0.754	**0.8**	0.427	**1.7**	0.249	**1.0**	0.967
NADP+	**2.4**	0.026	**1.1**	0.498	**1.1**	0.108	**2.7**	0.163	**1.5**	0.121

NADPH	**0.7**	0.814	**4.1**	0.272	**1.5**	0.459	**2.7**	0.268	**0.7**	0.394
FAD	**1.3**	0.336	**1.1**	0.492	**0.9**	0.740	**2.6**	0.193	**1.2**	0.115
Acetyl-CoA	**0.8**	0.612	**2.2**	0.154	**0.9**	0.690	**1.3**	0.535	**0.4**	0.074

				LOG TRANSFORMED DATA FOR EXP 3			
	EXPONENTIAL PHASE			EARLY STATIONARY		LATE STATIONARY	
	Exp 1			**FC (log2)**	PV (-log10)	**FC (log2)**	PV (-log10)
cAMP	**1.9**	0.239		**1.1**	1.456	**0.7**	2.301
AICARs	**114.4**	0.023		**7.4**	1.102	**2.9**	1.632
AICAR	**65.0**	0.007		**7.7**	1.770	**5.0**	2.125
Pantothenate	**1.1**	0.187		**-1.3**	1.420	**-2.6**	3.427
Pyruvate	**0.7**	0.414		**0.6**	0.879	**-0.2**	0.173
Ala	**3.6**	0.133		**-1.7**	0.462	**-1.2**	0.356
Lactate	**0.4**	0.136		**ND**		**0.6**	0.582
2-Oxobutanoate	**1.8**	0.169		**0.1**	0.063	**-0.3**	0.206
Dimethylglycine/4-Aminobutyrate	**0.9**	0.633		**-1.3**	0.967	**-1.1**	1.948
Ser	**0.2**	0.017		**-2.3**	2.000	**-1.2**	0.512
Glycerate	**1.4**	0.340		**0.3**	1.357	**0.2**	0.139
Uracil	**0.5**	0.577		**0.3**	0.301	**0.4**	2.069
Creatinine	**0.7**	0.303		**-0.5**	0.352	**0.4**	0.231
Pro	**1.8**	0.049		**0.3**	0.218	**0.4**	0.307
Fumarate[1]	**1.3**	0.131		**0.8**	0.807	**0.1**	0.538
Fumarate[2]	**0.5**	0.039		**-0.7**	0.256	**-1.5**	0.478
2-Ketoisovalerate (KIV)[1]	**0.9**	0.611		**0.1**	0.684	**0.1**	0.425
2-Ketoisovalerate (KIV)[2]	**1.5**	0.068		**1.2**	0.666	**0.0**	0.221
Guanidoacetic acid	**2.6**	0.159		**3.2**	1.699	**2.2**	0.848
Indole[1]	**2.1**	0.097		**2.4**	1.602	**2.8**	1.263
Indole[2]	**2.3**	0.467		**0.8**	0.453	**1.3**	1.197
Betaine/Val	**2.4**	0.004		**2.3**	4.568	**1.9**	1.987
Methylmalonic acid	**0.4**	0.068		**0.1**	0.032	**0.2**	0.146
Homoserine/Thr	**1.8**	0.008		**-0.2**	0.164	**-0.1**	0.110
3-S-Methylthiopropionate	**0.2**	0.081		**0.8**	0.812	**0.7**	1.131
Nicotinamide	**1.0**	0.928		**0.6**	0.299	**0.7**	0.528
Nicotinate	**0.8**	0.198		**0.0**	0.025	**0.2**	0.141
Thymine	**0.9**	0.684		**1.2**	2.222	**0.3**	0.658
Pyroglutamic acid (5-Oxoproline)[1]	**1.1**	0.568		**-0.5**	0.413	**-1.2**	2.148
Pyroglutamic acid (5-Oxoproline)[2]	**0.3**	0.274		**0.8**	0.697	**-0.6**	0.303
Citraconic acid	**1.1**	0.660		**-0.2**	0.360	**-0.2**	1.451
Hydroxyproline	**0.4**	0.034		**-1.3**	1.357	**-1.4**	2.534
Citraconic acid	**0.9**	0.489		**-0.2**	0.150	**-0.8**	2.003
Leu/Ile	**2.1**	0.046		**2.1**	2.699	**1.5**	2.013
Asn	**2.2**	0.013		**-0.7**	0.457	**-0.3**	0.361
Hydroxyisocaproate[1]	**1.0**	0.980		**-0.3**	1.284	**-0.4**	1.433
Hydroxyisocaproate[2]	**0.8**	0.213		**4.3**	1.699	**2.6**	2.745
Ornithine	**0.2**	0.002		**ND**		**ND**	
Asp	**1.0**	0.942		**-1.3**	0.963	**-1.4**	1.870
Malate[1]	**0.6**	0.029		**-1.3**	0.506	**-2.7**	0.750
Malate[2]	**0.6**	0.029		**-1.3**	0.506	**-3.0**	0.773
Homocysteine	**0.9**	0.448		**1.1**	0.917	**ND**	
Adenine[1]	**0.9**	0.658		**-0.3**	0.542	**0.2**	0.231
Adenine[2]	**0.8**	0.429		**0.1**	0.221	**1.0**	2.488
Hypoxanthine	**1.0**	0.994		**-0.3**	0.860	**-0.5**	3.033
p-Hydroxybenzoate	**0.7**	0.172		**-0.3**	0.272	**-0.4**	1.574

Acetyl-P	**0.3**	0.010	**-0.5**	0.498	**-4.0**	1.500
Carbamoyl-P	**1.1**	0.830	**2.7**	1.377	**-1.2**	1.040
a-Ketoglutarate	**0.8**	0.207	**1.0**	1.638	**0.9**	0.903
Gln	**1.3**	0.324	**0.1**	0.240	**-0.5**	0.292
Lys	**2.0**	0.309	**0.6**	0.426	**-0.4**	0.323
Glu[1]	**1.0**	0.938	**-0.3**	0.432	**-1.1**	2.190
Glu[2]	**0.7**	0.314	**1.3**	2.523	**-0.1**	0.286
2-Hydroxy-2-methylbutanedioic acid	**0.4**	0.022	**-0.5**	0.680	**-0.8**	1.821
Met	**0.9**	0.872	**-2.3**	1.276	**-0.7**	1.287
3-Methylphenylacetic acid	**1.0**	0.864	**-0.2**	0.139	**0.2**	0.222
Guanine	**0.7**	0.469	**0.9**	1.292	**0.5**	2.965
2,3-Dihydroxybenzoic acid	**5.1**	0.048	**3.8**	2.097	**1.9**	0.356
His	**2.0**	0.070	**-0.5**	0.604	**-0.8**	0.234
Orotate	**2.0**	0.043	**0.9**	1.959	**-1.1**	0.242
Dihydroorotate	**1.3**	0.126	**1.6**	0.747	**0.3**	0.754
Aminoadipate	**2.0**	0.045	**1.4**	3.000	**1.0**	1.672
Carnitine	**0.9**	0.407	**0.0**	0.105	**-0.1**	0.261
Phenylpyruvate	**1.3**	0.106	**0.6**	0.996	**-0.1**	0.106
Methionine sulfoxide	**1.0**	0.925	**-1.7**	0.516	**0.3**	0.096
Phe	**2.5**	0.003	**1.5**	1.699	**-0.3**	0.559
Atrolactic acid	**1.0**	0.959	**1.9**	2.523	**0.5**	1.183
Phenyllactic acid	**1.0**	0.959	**1.9**	2.523	**0.5**	1.183
Phosphoenolpyruvate (PEP)	**0.8**	0.287	**1.3**	0.690	**-1.4**	2.006
Uric acid	**14.7**	0.392	**ND**		**ND**	
Pyridoxamine	**1.3**	0.088	**0.3**	0.556	**-0.1**	0.083
D-Glyceraldehdye-3-P	**0.7**	0.116	**2.2**	1.770	**0.8**	0.888
sn-Glycerol-3-P	**0.7**	0.136	**0.1**	0.171	**-1.4**	0.667
Aconitate	**0.8**	0.112	**-0.3**	0.311	**-0.8**	1.300
Shikimate	**0.5**	0.053	**0.8**	1.260	**0.6**	1.815
N-Acetyl-L-ornithine	**0.5**	0.035	**-3.3**	1.046	**-2.8**	0.989
Arg	**3.0**	0.065	**-0.2**	0.141	**0.0**	0.043
Citrulline	**0.4**	0.018	**-1.3**	0.967	**0.9**	1.443
Ascorbic acid	**0.9**	0.694	**-0.2**	0.124	**-0.1**	0.297
N-Carbamoyl-L-aspartate	**0.7**	0.032	**0.8**	1.194	**-0.5**	1.731
Allantoate	**0.5**	0.562	**-0.2**	0.075	**0.1**	0.029
Isopropylmalate	**0.1**	0.050	**-3.3**	1.137	**-0.8**	1.170
Pyrophosphate	**0.9**	0.503	**0.9**	1.523	**0.0**	0.209
Gluconolactone	**0.7**	0.650	**-0.5**	1.056	**0.1**	0.080
Glucosamine	**1.2**	0.725	**-0.3**	0.498	**-0.2**	0.235
Hydroxyphenylpyruvate	**0.7**	0.225	**0.7**	1.553	**0.2**	0.589
Myo-inositol	**0.9**	0.606	**-0.7**	0.799	**-0.3**	0.979
Tyr	**2.8**	0.175	**0.8**	1.161	**-0.3**	0.814
4-Pyridoxic acid	**1.2**	0.423	**0.1**	0.340	**-1.2**	1.524
Phosphoserine	**0.2**	0.026	**-3.3**	1.638	**0.2**	0.121
Phosphoglycerate	**0.5**	0.022	**0.8**	0.658	**-1.0**	1.965
N-Acetyl-glutamine	**0.5**	0.060	**-0.2**	0.267	**-1.4**	1.739
Acetyllysine	**0.9**	0.660	**-2.3**	2.523	**-1.5**	0.731
Kynurenic acid	**0.9**	0.304	**1.8**	1.796	**1.2**	1.528
N-Acetyl-glutamate[1]	**0.1**	0.023	**0.7**	1.699	**-0.1**	0.347
N-Acetyl-glutamate[2]	**0.9**	0.462	**0.9**	1.678	**-0.5**	0.693
Citrate	**1.0**	0.821	**-2.3**	0.815	**-1.7**	2.866
2-Dehydro-D-gluconate	**0.8**	0.714	**0.6**	2.000	**0.3**	0.551
D-Gluconate	**0.7**	0.531	**2.5**	0.542	**-0.3**	0.070

Trp	**1.4**	0.057	**0.0**	0.058	**-0.1**	0.297
Acetylcarnitine	**ND**		**ND**		**ND**	
Xanthurenic acid	**2.1**	0.135	**0.8**	0.292	**0.3**	0.317
Lipoate	**0.3**	0.104	**-0.5**	0.303	**0.8**	0.352
Kynurenine	**0.5**	0.266	**0.5**	0.450	**0.0**	0.002
D-Glucarate[1]	**1.0**	0.941	**ND**		**ND**	
D-Glucarate[2]	**1.7**	0.114	**0.0**	0.043	**-0.3**	0.863
Deoxyribose-P	**0.4**	0.000	**0.7**	0.245	**0.0**	0.021
Cystathionine	**0.4**	0.043	**ND**		**ND**	
Prephenate	**0.6**	0.405	**1.2**	2.222	**0.4**	0.203
Deoxyuridine	**1.0**	0.981	**0.3**	0.680	**0.1**	0.061
Ribose-P	**1.1**	0.355	**5.2**	1.222	**0.6**	0.988
Thymidine	**1.6**	0.497	**ND**		**ND**	
Cytidine	**1.1**	0.791	**0.7**	0.382	**4.6**	0.428
Uridine	**0.9**	0.701	**5.2**	3.000	**2.3**	2.010
Deoxyinosine	**0.2**	0.300	**1.8**	0.903	**-1.2**	0.541
Shikimate-3-P	**0.2**	0.026	**0.8**	1.092	**1.0**	2.414
D-Gluconolactone-6-P	**0.6**	0.035	**-0.2**	0.270	**0.1**	0.082
Glycerophosphocholine	**1.0**	0.988	**0.3**	0.336	**-0.4**	0.482
D-Glucosamine-1-P	**1.6**	0.300	**1.5**	2.523	**1.1**	1.277
Glucose-1-P	**0.7**	0.021	**-0.5**	0.979	**-0.6**	0.622
Thiamine	**0.9**	0.916	**ND**		**ND**	
2,3-Diphosphoglyceric acid[1]	**0.5**	0.196	**1.3**	0.428	**-1.0**	0.174
2,3-Diphosphoglyceric acid[2]	**0.7**	0.471	**1.1**	0.863	**-0.2**	0.151
S-Ribosylhomocysteine (SRH)	**1.7**	0.060	**1.6**	3.000	**1.9**	2.014
Adenosine	**0.8**	0.561	**0.8**	2.222	**0.6**	1.263
Inosine	**1.3**	0.447	**1.5**	0.943	**2.7**	1.147
6-Phosphogluconate	**0.3**	0.004	**-0.2**	0.127	**-0.5**	0.659
1-Methyladenosine[1]	**1.4**	0.655	**ND**		**-1.2**	0.334
1-Methyladenosine[2]	**0.6**	0.133	**0.3**	0.397	**-0.3**	0.356
Guanosine	**2.1**	0.185	**ND**		**-1.1**	1.527
Xanthosine	**6.3**	0.001	**1.4**	2.523	**1.7**	1.748
Argininosuccinate	**0.3**	0.011	**-0.7**	0.472	**-0.8**	0.562
N-Acetyl-glucosamine	**0.8**	0.254	**-0.2**	0.152	**-0.6**	0.657
Glutathione	**1.2**	0.123	**-0.3**	0.216	**-0.3**	0.342
dUMP	**5.6**	0.031	**3.3**	1.886	**0.1**	0.081
Octoluse 8,1-P	**0.4**	0.015	**0.9**	0.664	**1.0**	1.394
dTMP	**0.7**	0.002	**0.4**	0.793	**-0.3**	0.707
CMP	**1.2**	0.195	**-0.7**	1.824	**-0.7**	1.203
UMP[1]	**1.4**	0.121	**-0.2**	0.112	**-0.7**	1.522
UMP[2]	**1.0**	0.818	**-0.3**	0.450	**-0.5**	0.515
dAMP	**1.2**	0.848	**1.4**	0.210	**-0.1**	0.127
Nicotinamide ribotide	**0.5**	0.025	**1.1**	2.699	**0.3**	0.173
Cellobiose	**0.9**	0.878	**-1.0**	1.328	**-0.7**	0.762
Trehalose/Sucrose	**0.9**	0.744	**-1.0**	1.328	**-0.7**	0.762
AMP	**1.9**	0.098	**-0.2**	0.313	**-0.1**	0.206
IMP[1]	**0.9**	0.745	**-1.3**	0.693	**ND**	
IMP[2]	**1.1**	0.830	**ND**		**ND**	
GMP	**1.6**	0.037	**-0.7**	0.815	**-1.5**	0.638
XMP	**1.4**	0.224	**0.9**	1.745	**-2.1**	0.336
Riboflavin	**1.5**	0.184	**0.6**	0.658	**-0.8**	1.597
S-Adenosylhomocysteine	**1.2**	0.421	**2.7**	3.701	**-1.3**	1.095

(SAH)						
dCDP	**2.4**	0.017	**-0.3**	0.564	**-0.3**	1.206
dTDP	**1.3**	0.408	**-0.2**	0.141	**-0.5**	1.141
CDP	**2.3**	0.148	**-1.0**	1.056	**-1.0**	0.946
UDP	**2.0**	0.212	**0.4**	0.839	**-0.7**	1.266
Trehalose-6-P	**ND**		**ND**		**1.8**	1.181
Adenylyl sulfate	**ND**		**0.9**	0.851	**0.9**	1.178
ADP	**2.0**	0.061	**0.3**	1.347	**-0.4**	1.054
GDP	**2.7**	0.005	**-1.0**	1.301	**-1.0**	1.382
Deoxycholic acid	**0.9**	0.212	**0.1**	0.959	**0.2**	0.197
FMN	**1.0**	0.951	**0.1**	0.138	**-0.4**	0.448
Cholesteryl sulfate	**1.0**	0.844	**-0.3**	2.097	**0.0**	0.091
dCTP	**2.0**	0.012	**-0.7**	1.284	**0.0**	0.064
dTTP	**1.4**	0.142	**-0.2**	0.248	**0.1**	0.070
CTP	**2.2**	0.040	**-1.3**	1.553	**-0.5**	0.402
UTP	**2.2**	0.023	**-1.0**	0.896	**0.0**	0.012
dATP	**1.9**	0.131	**-0.2**	0.336	**-0.1**	0.490
ATP	**2.7**	0.005	**-1.0**	1.301	**-0.1**	0.083
GTP	**1.9**	0.161	**-1.0**	1.921	**-0.1**	0.043
UDP-D-glucose	**1.8**	0.010	**1.0**	0.724	**-1.1**	0.369
UDP-D-glucuronate	**0.8**	0.491	**1.0**	3.000	**-2.6**	2.893
UDP-N-acetyl-glucosamine	**0.6**	0.219	**0.4**	0.451	**-0.1**	0.153
Glutathione disulfide	**1.0**	0.892	**0.6**	1.086	**1.4**	1.327
NAD+	**1.1**	0.104	**0.0**	0.008	**0.0**	0.023
NADH	**9.2**	0.065	**0.7**	1.222	**-0.4**	0.369
NADP+	**0.8**	0.426	**0.6**	1.208	**0.1**	0.966
NADPH	**6.2**	0.355	**-0.2**	0.154	**0.5**	0.338
FAD	**1.2**	0.707	**1.3**	1.770	**-0.1**	0.131
Acetyl-CoA	**1.1**	0.656	**-1.0**	0.947	**-0.1**	0.161

Early and late stationary phase data from Experiment 3 was plotted in Figure 1.
[1, 2] Multiple species with identical m/z.

TABLE S3. AICAR perturbs various areas of metabolism.

		EARLY STATIONARY PHASE						LATE STATIONARY PHASE					
		Linear Fold Changes			Log2 Transformed FC			Linear Fold Changes			Log2 Transformed FC		
		Exp 1	Exp 2	Exp 3	Exp 1	Exp 2	Exp 3	Exp 1	Exp 2	Exp 3	Exp 1	Exp 2	Exp 3
Carbon	cAMP	1.2	2.2	2.2	0.3	1.1	1.1	2.4	1.3	1.6	1.3	0.3	0.7
	Acetyl-P	0.2	0.7	0.7	-2.7	-0.5	-0.6	0.1	0.1	0.1	-3.1	-3.1	-4.0
	Glyceraldehdye-3-P	3.6	1.7	4.5	1.8	0.8	2.2	0.1	2.3	1.7	-3.1	1.2	0.8
	2,3-Diphosphoglycerate	6.8	1.8	2.5	2.8	0.9	1.3	2.9	0.8	0.5	1.5	-0.3	-1.0
	Aconitate	0.8	0.4	0.8	-0.4	-1.3	-0.3	0.8	0.4	0.6	-0.4	-1.3	-0.8
	2-Ketoglutarate	1.3	1.2	2.0	0.4	0.3	1.0	1.1	0.4	1.8	0.1	-1.3	0.9
	Fumarate[1]	1.3	1.3	1.7	0.3	0.4	0.8	1.9	0.9	1.1	0.9	-0.2	0.1
	Fumarate[2]	0.9	0.1	0.6	-0.2	-3.3	-0.6	0.2	0.6	0.3	-2.6	-0.7	-1.5
	Malate	0.8	0.1	0.4	-0.4	-3.5	-1.4	0.1	0.3	0.2	-3.7	-1.6	-2.7
	Citraconic acid	0.8	0.5	0.9	-0.4	-1.0	-0.2	0.8	0.4	0.6	-0.4	-1.2	-0.8
	Gluconolactone	0.4	1.0	0.7	-1.4	0.0	-0.5	2.5	1.4	1.0	1.3	0.5	0.1
	2-Dehydrogluconate	1.8	2.4	1.5	0.8	1.3	0.6	1.0	1.4	1.2	0.0	0.4	0.3
	Glucosamine-1-P	2.1	2.8	2.8	1.1	1.5	1.5	0.9	1.6	2.2	-0.1	0.7	1.1
	UDP-Glucuronate	5.7	ND	2.0	2.5	ND	1.0	0.1	ND	0.2	-3.0	ND	-2.6
	Atrolactic acid	1.7	1.5	3.7	0.8	0.6	1.9	1.0	1.5	1.4	-0.1	0.6	0.5
Cofactor	Pantothenate	0.3	0.2	0.4	-1.6	-2.1	-1.5	0.8	0.2	0.2	-0.3	-2.0	-2.6
	Dihydroxybenzoic acid	4.3	13.6	14.4	2.1	3.8	3.8	1.0	1.6	3.7	0.0	0.7	1.9
	Lipoate	0.3	0.4	0.7	-1.7	-1.4	-0.5	66.9	2.8	1.7	6.1	1.5	0.8
	S-Adenosylhomocysteine (SAH)	1.0	1.2	6.3	0.0	0.2	2.6	0.7	0.2	0.4	-0.5	-2.3	-1.3
	FAD	2.4	2.0	2.4	1.3	1.0	1.3	1.3	1.1	0.9	0.4	0.2	-0.1
	Nicotinamide ribotide (NMN)	1.4	2.8	2.1	0.5	1.5	1.0	1.8	2.0	1.3	0.9	1.0	0.3
Amino Acids	Glutamine	0.9	1.1	1.1	-0.1	0.1	0.2	0.4	0.9	0.7	-1.5	-0.2	-0.5
	Glu[1]	0.9	0.7	0.8	-0.2	-0.5	-0.4	0.3	0.5	0.5	-1.7	-1.0	-1.1
	Glu[2]	1.8	1.2	2.4	0.8	0.2	1.3	0.6	1.2	0.9	-0.8	0.3	-0.1
	Phosphoserine	0.1	0.0	0.1	-3.2	-4.9	-3.9	0.4	5.1	1.1	-1.3	2.3	0.2
	Ser	0.4	0.7	0.2	-1.2	-0.5	-2.2	0.8	1.4	0.4	-0.2	0.5	-1.2
	2-Ketoisovalerate (KIV)	1.2	1.2	1.1	0.3	0.3	0.2	0.7	1.5	1.1	-0.5	0.6	0.1
	Isopropylmalic acid	0.2	0.1	0.1	-2.3	-4.1	-3.5	3.5	4.1	0.6	1.8	2.0	-0.8
	Leu/Ile	1.9	3.8	4.4	0.9	1.9	2.1	1.1	1.1	2.9	0.2	0.1	1.5
	Hydroxyisocaproic acid	2.4	1.9	20.2	1.2	0.9	4.3	1.0	1.3	6.2	0.0	0.3	2.6
	Aminoadipic acid	2.5	2.5	2.7	1.3	1.3	1.4	1.1	1.8	2.0	0.1	0.8	1.0
	Acetyllysine	0.1	0.3	0.2	-3.3	-1.7	-2.2	0.2	0.2	0.3	-2.5	-2.2	-1.5
	Prephenate	5.0	2.6	2.3	2.3	1.4	1.2	0.9	0.6	1.3	-0.1	-0.7	0.4
	Phenylpyruvate	4.0	2.4	1.5	2.0	1.3	0.5	0.2	1.3	1.0	-2.1	0.3	-0.1
	Phe	13.4	6.9	2.9	3.7	2.8	1.5	0.4	0.7	0.8	-1.4	-0.6	-0.3
	Phenyllactic acid	1.8	1.5	3.7	0.8	0.6	1.9	1.0	1.5	1.4	-0.1	0.6	0.5
	Indole	2.6	3.2	5.1	1.4	1.7	2.3	2.3	4.1	7.1	1.2	2.0	2.8
	Kynurenic acid	4.0	1.5	3.5	2.0	0.6	1.8	1.6	0.6	2.3	0.6	-0.7	1.2
	Asp	0.8	0.3	0.4	-0.3	-1.7	-1.3	0.3	0.3	0.4	-1.8	-1.7	-1.4
	Asn	1.0	0.6	0.6	0.1	-0.8	-0.7	2.0	1.1	0.8	1.0	0.2	-0.3
	Met	0.5	0.5	0.2	-0.9	-1.0	-2.4	0.9	1.3	0.6	-0.2	0.4	-0.7
	S-Ribosylhomocysteine (SRH)	1.7	1.2	3.1	0.7	0.3	1.6	1.8	1.6	3.7	0.9	0.7	1.9
	N-Acetylornithine	0.0	0.0	0.1	-6.8	-4.9	-4.1	0.1	0.2	0.1	-3.7	-2.1	-2.8
	Carbamoyl-P	2.2	3.5	6.5	1.1	1.8	2.7	0.1	0.5	0.4	-3.3	-0.9	-1.2
	Citrulline	0.3	0.7	0.4	-1.5	-0.6	-1.3	1.1	1.1	1.9	0.1	0.2	0.9
	Argininosuccinate	0.9	0.3	0.6	-0.1	-2.0	-0.7	0.2	0.4	0.6	-2.7	-1.3	-0.8
	Arg	2.2	1.3	0.9	1.1	0.4	-0.2	0.4	0.4	1.0	-1.2	-1.4	0.0
	Betaine	2.5	2.9	5.0	1.3	1.5	2.3	1.9	2.3	3.6	0.9	1.2	1.9
	Dimethylglycine/4-Aminobutyrate	0.3	0.6	0.4	-1.6	-0.8	-1.2	0.5	0.5	0.5	-1.0	-1.1	-1.1

	Hydroxyproline	0.2	0.1	<u>0.4</u>	-2.5	-2.9	-1.4	0.2	<u>0.5</u>	<u>0.4</u>	-2.1	-1.1	-1.4
	Pyroglutamic acid (5-Oxoproline)[1]	0.9	0.8	0.7	-0.2	-0.4	-0.4	0.2	<u>0.4</u>	<u>0.4</u>	-2.1	-1.2	-1.2
	Pyroglutamic acid (5-Oxoproline)[2]	1.1	3.9	1.7	0.1	2.0	0.7	0.7	0.7	0.7	-0.6	-0.5	-0.6
	Guanidoacetic acid	1.7	1.6	<u>8.9</u>	0.7	0.7	3.1	2.2	2.2	4.7	1.1	1.1	2.2
Purines	AICARs	407.2	<u>94.4</u>	164.3	8.7	6.6	7.4	<u>2.8</u>	3.4	<u>7.4</u>	1.5	1.8	2.9
	AICAR	<u>346.6</u>	<u>367.2</u>	<u>206.0</u>	8.4	8.5	7.7	16.8	<u>34.1</u>	<u>32.7</u>	4.1	5.1	5.0
	Adenine	<u>0.5</u>	0.7	0.8	-0.9	-0.4	-0.3	1.1	<u>1.5</u>	1.2	0.1	0.6	0.2
	Hypoxanthine	0.5	1.1	0.8	-1.0	0.2	-0.3	<u>0.5</u>	0.5	<u>0.7</u>	-1.1	-1.1	-0.5
	Adenosine	1.4	2.7	<u>1.8</u>	0.5	1.4	0.9	1.1	1.3	1.5	0.2	0.3	0.6
	Inosine	<u>2.5</u>	1.1	2.9	1.3	0.2	1.5	1.7	1.2	6.4	0.8	0.3	2.7
	Guanosine	ND	0.3	ND	ND	-1.8	ND	0.9	0.9	<u>0.5</u>	-0.1	-0.1	-1.1
	Xanthosine	4.8	4.9	<u>2.6</u>	2.3	2.3	1.4	2.7	<u>4.2</u>	<u>3.3</u>	1.5	2.1	1.7
	AMP	<u>1.4</u>	1.2	0.9	0.4	0.2	-0.1	1.5	0.6	0.9	0.6	-0.7	-0.1
	XMP	3.8	1.3	<u>1.9</u>	1.9	0.3	0.9	ND	0.4	0.2	ND	-1.3	-2.1
	ADP	4.1	2.0	<u>1.2</u>	2.0	1.0	0.3	1.0	0.9	0.8	0.1	-0.2	-0.4
	dATP	0.9	1.5	0.9	-0.1	0.5	-0.2	0.7	1.5	0.9	-0.6	0.6	-0.1
Pyrimidines	Dihydroorotate	5.7	<u>5.1</u>	3.1	2.5	2.3	1.6	0.8	1.7	1.2	-0.4	0.8	0.3
	Orotate	2.7	1.9	<u>1.9</u>	1.4	1.0	0.9	3.2	1.4	0.5	1.7	0.5	-1.1
	UMP	1.2	1.0	0.9	0.3	-0.1	-0.1	0.9	0.6	<u>0.6</u>	-0.2	-0.9	-0.7
	UMP	1.1	0.8	0.8	0.1	-0.3	-0.4	0.8	<u>0.6</u>	0.7	-0.3	-0.7	-0.5
	Uracil	0.5	0.6	1.2	-0.9	-0.7	0.3	1.1	0.9	<u>1.3</u>	0.2	-0.2	0.4
	Uridine	3.0	<u>12.1</u>	<u>37.4</u>	1.6	3.6	5.2	0.6	0.6	<u>5.0</u>	-0.7	-0.8	2.3
	Cytidine	1.5	<u>9.7</u>	1.6	0.6	3.3	0.6	5.9	10.9	24.0	2.6	3.4	4.6
	CMP	0.7	0.4	<u>0.6</u>	-0.5	-1.3	-0.8	<u>1.9</u>	<u>0.5</u>	0.6	0.9	-1.0	-0.7
	CDP	1.1	<u>0.3</u>	0.5	0.1	-1.7	-1.1	1.3	0.5	0.5	0.4	-1.0	-1.0
	CTP	1.0	0.5	<u>0.4</u>	0.0	-0.9	-1.4	0.6	<u>1.5</u>	0.7	-0.7	0.6	-0.5
	dUMP	<u>14.3</u>	<u>22.2</u>	<u>9.8</u>	3.8	4.5	3.3	2.7	0.9	1.1	1.4	-0.2	0.1
	Thymine	2.2	3.4	<u>2.3</u>	1.1	1.8	1.2	0.7	0.8	1.2	-0.6	-0.4	0.3

Early stationary phase data was plotted in Figure 4.
Underlined fold changes indicate instances where p-value was <0.05.
In instances where differences were not detected (or significant) in early stationary phase, data were included if statistically significant differences were present in late stationary phase.
[1, 2] Multiple species with identical m/z.

TABLE S4. Metabolome of *purH* and *icc* mutants

	EARLY STATIONARY PHASE							
	Exp 1				Exp 2			
	purH		*icc*		*purH*		*icc*	
Metabolite	**Fold Change**	p-value	**Fold Change**	p-value	**Fold Change**	p-value	**Fold Change**	p-value
cAMP	**2.2**	0.165	**2.7**	0.293	**2.2**	0.035	**3.3**	0.004
AICARs	**ND**	-	**ND**	-	**169.1**	0.079	**0.3**	0.354
AICAR	**367.2**	0.003	**1.0**	0.943	**206.0**	0.017	**0.9**	0.680
Pantothenate	**0.2**	0.002	**0.6**	0.367	**0.4**	0.038	**0.2**	0.008
Lactate	**ND**	-	**ND**	-	**0.6**	0.493	**0.4**	0.371
Dimethylglycine/4-Aminobutyrate	**1.5**	0.375	**0.6**	0.224	**ND**	-	**ND**	-
Ser	**0.7**	0.454	**0.5**	0.105	**0.2**	0.006	**0.9**	0.652
Glycerate	**1.1**	0.685	**0.8**	0.266	**1.2**	0.044	**1.3**	0.650
Uracil	**0.6**	0.550	**0.3**	0.002	**1.2**	0.500	**1.4**	0.393
Pro	**1.4**	0.386	**0.5**	0.181	**1.2**	0.605	**0.1**	0.135
Fumarate[1]	**1.3**	0.011	**ND**	-	**1.7**	0.156	**1.3**	0.061
Fumarate[2]	**0.1**	0.014	**0.2**	0.010	**0.6**	0.554	**0.1**	0.163
2-Ketoisovalerate (KIV)	**1.5**	0.198	**0.2**	0.002	**ND**	-	**ND**	-
Guanidoacetic acid	**1.6**	0.449	**0.2**	0.056	**8.9**	0.020	**0.4**	0.014
Indole	**ND**	-	**ND**	-	**5.1**	0.025	**0.4**	0.084
Phenylacetonitrile	**3.2**	0.009	**0.3**	0.129	**ND**	-	**ND**	-
Betaine/Val	**2.9**	0.113	**0.4**	0.027	**5.0**	0.000	**0.5**	0.025
Succinate	**0.2**	0.034	**0.2**	0.030	**1.1**	0.930	**0.3**	0.194
Homoserine/Thr	**1.2**	0.099	**0.5**	0.113	**0.9**	0.685	**0.7**	0.383
3-S-Methylthiopropionate	**1.8**	0.534	**0.4**	0.002	**1.8**	0.154	**0.6**	0.302
Nicotinamide	**1.9**	0.125	**1.6**	0.649	**1.9**	0.218	**1.2**	0.603
Nicotinate	**0.8**	0.300	**0.7**	0.118	**1.0**	0.990	**2.6**	0.004
Thymine	**3.4**	0.445	**0.4**	0.167	**2.3**	0.006	**2.3**	0.253
Pyroglutamic acid (5-Oxoproline)[1]	**0.8**	0.268	**0.6**	0.334	**0.7**	0.386	**0.5**	0.153
Pyroglutamic acid (5-Oxoproline)[2]	**3.9**	0.172	**1.0**	0.964	**1.7**	0.201	**ND**	-
Citraconic acid	**0.5**	0.062	**ND**	-	**0.9**	0.708	**0.5**	0.206
N-Acetylalanine	**0.1**	0.158	**0.3**	0.233	**0.4**	0.044	**2.0**	0.099
Leu/Ile	**3.0**	0.170	**0.5**	0.108	**4.0**	0.006	**1.2**	0.615
Oxaloacetate	**1.2**	0.477	**0.7**	0.320	**ND**	-	**ND**	-
Asn	**0.6**	0.031	**0.4**	0.062	**0.6**	0.349	**0.6**	0.341
Hydroxyisocaproate	**1.9**	0.314	**0.9**	0.743	**20.2**	0.020	**1.3**	0.155
Asp	**0.3**	0.017	**0.4**	0.035	**0.4**	0.109	**0.5**	0.150
Malate	**0.0**	0.040	**0.1**	0.041	**0.4**	0.305	**0.0**	0.174
Homocysteine	**1.1**	0.838	**0.1**	0.192	**ND**	-	**ND**	-
Adenine[1]	**0.7**	0.149	**0.4**	0.014	**0.8**	0.287	**0.7**	0.338
Adenine[2]	**1.3**	0.710	**ND**	-	**1.1**	0.601	**0.3**	0.018
Hypoxanthine	**1.1**	0.826	**1.0**	0.899	**0.8**	0.138	**1.2**	0.277
p-Hydroxybenzoate	**2.4**	0.341	**1.3**	0.440	**0.8**	0.534	**0.9**	0.671
Acetyl-P	**0.7**	0.255	**0.6**	0.354	**0.7**	0.318	**0.7**	0.496
Carbamoyl-P	**3.5**	0.155	**1.3**	0.628	**6.5**	0.042	**2.0**	0.307
a-Ketoglutarate	**1.2**	0.289	**0.3**	0.003	**2.0**	0.023	**0.8**	0.388
Gln	**1.1**	0.755	**1.3**	0.659	**1.1**	0.576	**0.7**	0.082
Lys	**0.9**	0.246	**0.7**	0.427	**1.5**	0.375	**7.6**	0.278

Glu[1]	**0.7**	0.137	**0.6**	0.291	**0.8**	0.370	**0.6**	0.135
Glu[2]	**1.2**	0.811	**1.1**	0.909	**2.4**	0.003	**4.8**	0.051
2-Hydroxy-2-methylbutanedioic acid	**0.5**	0.254	**0.5**	0.171	**0.7**	0.209	**1.2**	0.272
Met	**0.5**	0.001	**0.2**	0.015	**0.2**	0.053	**0.3**	0.063
Guanine	**2.8**	0.444	**0.9**	0.700	**1.9**	0.051	**1.6**	0.199
Xanthine	**1.4**	0.617	**0.2**	0.006	**0.8**	0.608	**1.0**	0.973
2-Hydroxyphenylacetate[1]	**1.6**	0.025	**1.1**	0.377	**ND**	-	**ND**	-
2-Hydroxyphenylacetate[2]	**0.9**	0.855	**0.8**	0.433	**ND**	-	**ND**	-
2,3-Dihydroxybenzoic acid	**13.6**	0.100	**1.5**	0.612	**14.4**	0.008	**1.0**	0.972
His	**4.2**	0.173	**3.5**	0.353	**ND**	-	**ND**	-
Orotate	**1.9**	0.171	**0.8**	0.354	**1.9**	0.011	**0.9**	0.625
Dihydroorotate	**5.1**	0.010	**1.9**	0.210	**3.1**	0.179	**2.1**	0.307
Allantoin	**ND**	-	**ND**	-	**14.2**	0.047	**0.9**	0.622
Aminoadipate	**2.5**	0.114	**1.1**	0.837	**2.7**	0.001	**1.4**	0.164
Phenylpyruvate[1]	**2.4**	0.012	**0.6**	0.273	**1.5**	0.101	**0.8**	0.186
Phenylpyruvate[2]	**3.1**	0.155	**4.2**	0.038	**2.3**	0.079	**2.9**	0.309
Methionine sulfoxide	**0.7**	0.147	**1.5**	0.536	**0.3**	0.305	**4.1**	0.213
Phe	**6.9**	0.083	**3.0**	0.226	**2.9**	0.020	**8.7**	0.314
Phenyllactic acid	**1.5**	0.395	**0.7**	0.328	**3.7**	0.003	**1.9**	0.246
Quinolinate	**ND**	-	**ND**	-	**0.1**	0.078	**0.8**	0.484
Phosphoenolpyruvate (PEP)	**0.6**	0.201	**0.7**	0.493	**2.5**	0.204	**0.3**	0.109
DHAP	**1.7**	0.144	**0.5**	0.147	**4.5**	0.017	**1.7**	0.105
sn-Glycerol-3-P	**1.3**	0.183	**0.7**	0.477	**1.1**	0.674	**0.1**	0.032
Aconitate	**0.4**	0.033	**0.5**	0.191	**0.8**	0.489	**0.4**	0.098
Shikimate	**ND**	-	**ND**	-	**1.7**	0.055	**0.7**	0.316
2-Isopropyl-3-oxosuccinate	**1.1**	0.929	**0.3**	0.123	**ND**	-	**ND**	-
N-Acetyl-L-ornithine	**0.0**	0.008	**0.1**	0.002	**0.1**	0.090	**0.3**	0.129
Arg	**1.3**	0.418	**0.7**	0.575	**1.1**	0.747	**6.3**	0.223
Citrulline	**0.7**	0.250	**0.4**	0.080	**0.4**	0.108	**0.2**	0.056
Ascorbic acid	**1.4**	0.269	**0.9**	0.692	**0.6**	0.228	**0.7**	0.362
N-Carbamoyl-L-aspartate	**15.6**	0.068	**2.6**	0.277	**1.7**	0.064	**1.5**	0.137
Isopropylmalate	**0.1**	0.005	**0.4**	0.080	**0.1**	0.073	**0.2**	0.098
Pyrophosphate	**1.8**	0.465	**0.8**	0.493	**1.9**	0.030	**1.0**	0.959
Gluconolactone	**1.0**	0.913	**0.9**	0.844	**0.7**	0.088	**0.8**	0.384
Glucosamine	**0.9**	0.772	**0.8**	0.345	**0.8**	0.318	**0.9**	0.579
Hydroxyphenylpyruvate	**1.1**	0.520	**0.8**	0.372	**1.6**	0.028	**0.4**	0.036
Myo-inositol	**0.7**	0.000	**0.3**	0.054	**0.6**	0.159	**0.5**	0.072
Tyr	**4.1**	0.128	**4.4**	0.306	**1.8**	0.069	**6.0**	0.317
Phosphoserine	**0.0**	0.030	**0.4**	0.079	**0.1**	0.023	**0.4**	0.033
Phosphoglycerate	**0.7**	0.389	**0.7**	0.469	**1.7**	0.220	**0.4**	0.008
N-Acetyl-glutamine	**0.7**	0.351	**2.0**	0.445	**0.9**	0.541	**0.9**	0.435
Acetyllysine	**0.3**	0.005	**1.0**	0.966	**0.2**	0.003	**3.6**	0.142
Kynurenic acid	**1.5**	0.489	**ND**	-	**3.5**	0.016	**1.0**	0.896
N-Acetyl-glutamate[1]	**1.1**	0.878	**0.8**	0.665	**1.6**	0.020	**1.0**	0.895
N-Acetyl-glutamate[2]	**1.0**	0.789	**2.7**	0.409	**1.9**	0.013	**23.0**	0.165
Citrate	**0.2**	0.070	**0.4**	0.116	**0.2**	0.153	**0.3**	0.208
2-Dehydro-D-gluconate	**2.4**	0.391	**ND**	-	**1.5**	0.010	**0.8**	0.142
D-Gluconate	**2.0**	0.486	**0.3**	0.412	**5.5**	0.287	**14.0**	0.415
D-Erythrose-4-P	**ND**	-	**ND**	-	**1.3**	0.103	**2.5**	0.054
Trp	**2.2**	0.132	**2.0**	0.392	**1.0**	0.875	**1.1**	0.805
D-Glucarate[1]	**0.7**	0.315	**0.6**	0.202	**1.0**	0.905	**1.1**	0.700

Deoxyribose-P	**0.6**	0.070	**0.4**	0.093	**1.6**	0.569	**0.6**	0.113
Prephenate	**ND**	_	**ND**	_	**2.3**	0.006	**2.5**	0.326
Deoxyuridine	**1.2**	0.529	**1.0**	0.884	**1.2**	0.209	**0.9**	0.603
Ribose-P	**5.0**	0.124	**1.1**	0.780	**1.7**	0.033	**1.4**	0.198
Thymidine	**0.2**	0.348	**0.5**	0.534	**ND**	_	**ND**	_
Ribose-P	**ND**	_	**ND**	_	**71.8**	0.058	**0.8**	0.628
Cytidine	**9.7**	0.029	**0.9**	0.879	**1.6**	0.415	**1.0**	0.962
Uridine	**12.1**	0.027	**ND**	_	**37.4**	0.001	**4.1**	0.452
Deoxyinosine	**0.6**	0.416	**0.3**	0.215	**3.7**	0.118	**ND**	_
Shikimate-3-P	**0.8**	0.840	**0.2**	0.187	**1.5**	0.035	**0.6**	0.057
D-Gluconolactone-6-P	**ND**	_	**ND**	_	**1.0**	0.822	**1.2**	0.307
D-Glucosamine-1-P	**2.5**	0.244	**0.8**	0.595	**2.8**	0.003	**1.8**	0.077
D-Fructose 6-phosphate	**3.0**	0.192	**1.4**	0.513	**ND**	_	**ND**	_
Glucose-P	**3.0**	0.192	**ND**	_	**0.7**	0.105	**1.1**	0.584
S-Ribosylhomocysteine (SRH)	**1.2**	0.306	**0.5**	0.102	**3.1**	0.001	**0.9**	0.763
Adenosine	**2.7**	0.237	**1.5**	0.118	**1.8**	0.006	**1.5**	0.070
Inosine	**1.1**	0.767	**1.2**	0.671	**2.9**	0.114	**0.9**	0.730
6-Phosphogluconate	**ND**	_	**ND**	_	**0.9**	0.746	**1.2**	0.512
Xanthosine	**4.9**	0.184	**1.0**	0.894	**2.6**	0.003	**0.6**	0.138
D-Sedoheptulose-1,7-P	**8.8**	0.168	**2.7**	0.318	**ND**	_	**ND**	_
Argininosuccinate	**0.3**	0.002	**0.3**	0.014	**0.6**	0.337	**0.6**	0.294
N-Acetyl-glucosamine-1,6-P	**0.7**	0.114	**0.4**	0.066	**ND**	_	**ND**	_
Glutathione	**0.9**	0.702	**0.0**	0.017	**0.8**	0.608	**0.0**	0.069
dCMP	**6.1**	0.025	**1.9**	0.336	**3.1**	0.021	**0.9**	0.834
dUMP	**22.2**	0.048	**2.7**	0.180	**8.9**	0.014	**1.0**	0.968
Octoluse 8,1-P	**0.9**	0.922	**1.2**	0.725	**1.9**	0.232	**1.1**	0.674
dTMP	**0.9**	0.766	**1.1**	0.823	**1.3**	0.161	**1.1**	0.654
CMP	**0.4**	0.061	**0.3**	0.033	**0.6**	0.015	**1.6**	0.284
UMP[1]	**1.0**	0.694	**1.0**	0.964	**0.9**	0.773	**2.7**	0.004
dAMP	**0.9**	0.691	**1.6**	0.589	**ND**	_	**ND**	_
Trehalose/Sucrose	**1.2**	0.045	**0.8**	0.669	**0.5**	0.047	**0.8**	0.147
Cellobiose	**ND**	_	**ND**	_	**0.7**	0.431	**0.8**	0.551
dGMP	**1.2**	0.275	**1.2**	0.777	**0.9**	0.486	**1.4**	0.066
IMP[2]	**1.9**	0.408	**0.1**	0.192	**0.4**	0.203	**0.3**	0.123
GMP	**0.7**	0.048	**1.0**	0.991	**0.6**	0.153	**1.2**	0.394
XMP	**1.3**	0.690	**2.4**	0.335	**1.9**	0.018	**0.4**	0.047
Riboflavin	**2.8**	0.402	**ND**	_	**1.5**	0.220	**1.2**	0.469
S-Adenosylhomocysteine (SAH)	**ND**	_	**ND**	_	**6.3**	0.000	**3.1**	0.036
dCDP	**ND**	_	**ND**	_	**0.5**	0.002	**1.6**	0.496
dTDP	**1.4**	0.537	**2.1**	0.344	**0.9**	0.723	**1.9**	0.162
CDP	**0.3**	0.012	**0.3**	0.006	**0.5**	0.088	**0.8**	0.497
UDP	**1.6**	0.556	**0.7**	0.601	**1.3**	0.145	**2.4**	0.059
Trehalose-6-P	**0.8**	0.597	**3.7**	0.466	**ND**	_	**0.5**	0.588
Adenylyl sulfate	**3.2**	0.242	**9.5**	0.033	**1.9**	0.141	**3.1**	0.028
ADP	**2.0**	0.156	**1.3**	0.546	**1.2**	0.045	**2.1**	0.167
GDP	**1.1**	0.823	**0.6**	0.207	**0.6**	0.006	**1.1**	0.578
FMN	**1.0**	0.973	**0.5**	0.102	**1.1**	0.728	**1.4**	0.303
dCTP	**1.0**	0.942	**0.3**	0.008	**0.6**	0.052	**1.4**	0.447
dTTP	**0.9**	0.234	**0.6**	0.229	**0.9**	0.565	**1.1**	0.812
CTP	**0.5**	0.203	**0.2**	0.010	**0.4**	0.028	**1.2**	0.664
UTP	**2.1**	0.502	**0.5**	0.198	**0.5**	0.127	**0.9**	0.887
dATP	**1.5**	0.518	**0.3**	0.181	**0.9**	0.461	**1.5**	0.033

ATP	**1.4**	0.607	**0.5**	0.330	**0.5**	0.050	**1.0**	0.983
GTP	**1.6**	0.613	**0.3**	0.024	**0.5**	0.012	**1.1**	0.878
UDP-D-glucose	**13.2**	0.086	**3.9**	0.239	**2.0**	0.189	**3.0**	0.107
UDP-D-glucuronate	**ND**	-	**ND**	-	**2.2**	0.003	**2.7**	0.250
UDP-N-acetyl-glucosamine	**5.0**	0.305	**0.9**	0.797	**1.3**	0.354	**2.1**	0.147
Glutathione disulfide	**0.9**	0.554	**0.4**	0.090	**1.5**	0.082	**0.1**	0.028
NAD+	**1.3**	0.110	**0.7**	0.499	**1.0**	0.982	**1.0**	0.809
NADH	**1.2**	0.892	**ND**	-	**1.6**	0.060	**1.9**	0.021
NADP+	**1.8**	0.274	**0.6**	0.356	**1.5**	0.062	**1.0**	0.915
	LATE STATIONARY PHASE							
	Exp 1				Exp 2			
	purH		*icc*		*purH*		*icc*	
cAMP	**1.3**	0.105	**1.5**	0.184	**1.6**	0.005	**2.2**	0.098
AICARs	**ND**	-	**ND**	-	**7.6**	0.023	**1.6**	0.300
AICAR	**34.1**	0.032	**2.8**	0.061	**32.7**	0.007	**0.6**	0.122
Pantothenate	**0.2**	0.000	**0.2**	0.000	**0.2**	0.000	**0.6**	0.128
Lactate	**ND**	-	**ND**	-	**1.0**	0.898	**0.9**	0.761
Dimethylglycine/4-Aminobutyrate	**0.4**	0.018	**1.5**	0.406	**ND**	-	**ND**	-
Ser	**1.4**	0.017	**1.6**	0.499	**0.4**	0.308	**6.6**	0.454
Glycerate	**1.0**	0.986	**1.1**	0.657	**1.1**	0.726	**1.2**	0.450
Uracil	**0.9**	0.296	**1.3**	0.198	**1.3**	0.009	**1.3**	0.357
Pro	**0.9**	0.742	**2.1**	0.080	**1.4**	0.375	**2.5**	0.180
Fumarate[1]	**ND**	-	**ND**	-	**1.1**	0.290	**1.1**	0.234
Fumarate[2]	**0.6**	0.078	**4.3**	0.184	**0.3**	0.332	**1.4**	0.596
2-Ketoisovalerate (KIV)	**3.8**	0.005	**0.7**	0.070	**ND**	-	**ND**	-
Guanidoacetic acid	**2.2**	0.233	**0.8**	0.617	**4.7**	0.142	**0.2**	0.188
Indole	**ND**	-	**ND**	-	**7.1**	0.055	**1.0**	0.897
Phenylacetonitrile	**4.1**	0.038	**1.1**	0.753	**ND**	-	**ND**	-
Betaine/Val	**2.3**	0.001	**0.8**	0.055	**3.6**	0.010	**0.5**	0.033
Succinate	**0.9**	0.051	**1.4**	0.033	**1.1**	0.715	**0.8**	0.680
Homoserine/Thr	**0.9**	0.536	**1.3**	0.175	**0.9**	0.776	**1.1**	0.742
3-S-Methylthiopropionate	**1.2**	0.597	**0.7**	0.323	**1.6**	0.074	**1.4**	0.390
Nicotinamide	**1.4**	0.320	**1.5**	0.207	**1.7**	0.297	**1.2**	0.560
Nicotinate	**0.9**	0.676	**1.1**	0.710	**1.6**	0.113	**2.0**	0.045
Thymine	**0.8**	0.433	**1.5**	0.616	**1.2**	0.220	**1.1**	0.872
Pyroglutamic acid (5-Oxoproline)[1]	**0.4**	0.012	**2.1**	0.120	**0.4**	0.007	**3.9**	0.165
Pyroglutamic acid (5-Oxoproline)[2]	**0.7**	0.221	**4.6**	0.031	**ND**	-	**ND**	-
Citraconic acid	**0.4**	0.064	**ND**	-	**0.6**	0.010	**1.9**	0.564
N-Acetylalanine	**0.5**	0.008	**1.1**	0.320	**0.4**	0.003	**1.7**	0.257
Leu/Ile	**1.0**	0.851	**1.0**	0.970	**3.1**	0.000	**0.7**	0.194
Oxaloacetate	**1.4**	0.494	**2.0**	0.038	**ND**	-	**ND**	-
Asn	**1.1**	0.644	**1.2**	0.331	**0.8**	0.435	**1.7**	0.034
Hydroxyisocaproate	**1.3**	0.089	**1.7**	0.263	**6.2**	0.002	**7.6**	0.299
Asp	**0.3**	0.001	**1.8**	0.000	**0.4**	0.013	**2.1**	0.088
Malate	**0.5**	0.096	**23.8**	0.082	**0.1**	0.169	**1.3**	0.602
Homocysteine	**1.5**	0.153	**ND**	-	**ND**	-	**ND**	-
Adenine[1]	**1.5**	0.038	**0.2**	0.011	**1.2**	0.588	**3.2**	0.339
Adenine[2]	**1.7**	0.027	**ND**	-	**1.9**	0.003	**1.2**	0.796
Hypoxanthine	**0.5**	0.081	**1.4**	0.376	**0.7**	0.001	**1.0**	0.405

p-Hydroxybenzoate	**0.6**	0.414	**1.0**	0.997	**0.8**	0.027	**0.9**	0.114
Acetyl-P	**0.1**	0.085	**5.9**	0.029	**0.1**	0.032	**7.2**	0.138
Carbamoyl-P	**0.5**	0.330	**2.3**	0.086	**0.4**	0.091	**1.6**	0.097
a-Ketoglutarate	**0.4**	0.056	**0.5**	0.066	**1.8**	0.125	**2.4**	0.305
Gln	**0.9**	0.591	**2.7**	0.134	**0.7**	0.511	**3.6**	0.230
Lys	**0.4**	0.004	**0.6**	0.017	**0.8**	0.476	**0.9**	0.762
Glu[1]	**0.5**	0.011	**2.0**	0.098	**0.5**	0.008	**3.7**	0.158
Glu[2]	**1.2**	0.785	**1.8**	0.040	**0.9**	0.518	**0.9**	0.647
2-Hydroxy-2-methylbutanedioic acid	**0.2**	0.060	**1.0**	0.992	**0.6**	0.015	**1.3**	0.431
Met	**1.3**	0.609	**0.6**	0.408	**0.6**	0.052	**1.0**	0.837
Guanine	**0.8**	0.091	**1.0**	0.975	**1.5**	0.001	**1.0**	0.907
Xanthine	**1.1**	0.725	**1.3**	0.434	**0.6**	0.137	**0.7**	0.502
2-Hydroxyphenylacetate[1]	**0.9**	0.054	**1.0**	0.908	**ND**	-	**ND**	-
2-Hydroxyphenylacetate[2]	**0.6**	0.003	**0.9**	0.322	**ND**	-	**ND**	-
2,3-Dihydroxybenzoic acid	**1.6**	0.560	**1.0**	0.981	**3.7**	0.441	**4.5**	0.241
His	**0.5**	0.186	**0.4**	0.143	**0.6**	0.257	**2.1**	0.075
Orotate	**1.4**	0.615	**13.2**	0.303	**0.5**	0.573	**0.9**	0.952
Dihydroorotate	**1.7**	0.312	**5.6**	0.242	**1.2**	0.176	**3.5**	0.297
Allantoin	**ND**	-	**ND**	-	**ND**	-	**ND**	-
Aminoadipate	**1.8**	0.005	**2.2**	0.012	**2.0**	0.021	**1.0**	0.753
Phenylpyruvate[1]	**1.3**	0.149	**1.2**	0.231	**1.0**	0.784	**0.9**	0.303
Phenylpyruvate[2]	**1.2**	0.594	**1.4**	0.186	**1.2**	0.823	**1.6**	0.451
Methionine sulfoxide	**5.8**	0.059	**8.0**	0.049	**1.2**	0.802	**1.1**	0.876
Phe	**0.7**	0.018	**1.7**	0.069	**0.8**	0.276	**0.7**	0.200
Phenyllactic acid	**1.5**	0.205	**1.0**	0.921	**1.4**	0.066	**0.9**	0.881
Quinolinate	**ND**	-	**ND**	-	**0.4**	0.033	**1.0**	0.918
Phosphoenolpyruvate (PEP)	**0.7**	0.320	**1.3**	0.266	**0.4**	0.010	**1.6**	0.149
DHAP	**2.3**	0.031	**4.4**	0.208	**1.7**	0.129	**1.9**	0.332
sn-Glycerol-3-P	**1.9**	0.045	**1.8**	0.093	**0.4**	0.215	**0.2**	0.162
Aconitate	**0.4**	0.021	**2.9**	0.029	**0.6**	0.050	**1.2**	0.793
Shikimate	**ND**	-	**ND**	-	**1.5**	0.015	**0.7**	0.135
2-Isopropyl-3-oxosuccinate	**1.0**	0.843	**0.7**	0.392	**ND**	-	**ND**	-
N-Acetyl-L-ornithine	**0.2**	0.168	**11.4**	0.113	**0.1**	0.103	**1.9**	0.442
Arg	**0.4**	0.011	**0.5**	0.033	**1.0**	0.906	**0.7**	0.247
Citrulline	**1.1**	0.638	**3.5**	0.239	**2.1**	0.007	**0.7**	0.215
Ascorbic acid	**0.5**	0.033	**1.2**	0.203	**1.2**	0.358	**1.7**	0.200
N-Carbamoyl-L-aspartate	**0.7**	0.232	**11.0**	0.395	**0.7**	0.019	**1.2**	0.443
Isopropylmalate	**4.1**	0.002	**0.4**	0.008	**0.6**	0.068	**0.4**	0.063
Pyrophosphate	**1.0**	0.858	**1.3**	0.638	**1.0**	0.618	**1.0**	0.734
Gluconolactone	**1.4**	0.237	**0.5**	0.120	**1.0**	0.832	**0.8**	0.602
Glucosamine	**0.6**	0.359	**1.2**	0.552	**0.9**	0.582	**1.6**	0.308
Hydroxyphenylpyruvate	**1.0**	0.804	**1.3**	0.050	**1.2**	0.258	**0.8**	0.122
Myo-inositol	**0.8**	0.380	**0.8**	0.507	**0.8**	0.105	**1.0**	0.770
Tyr	**0.7**	0.081	**1.4**	0.040	**0.8**	0.154	**1.0**	0.927
Phosphoserine	**5.1**	0.048	**0.7**	0.376	**1.1**	0.756	**0.7**	0.568
Phosphoglycerate	**1.0**	0.953	**1.2**	0.498	**0.5**	0.011	**1.7**	0.259
N-Acetyl-glutamine	**0.2**	0.016	**3.2**	0.084	**0.4**	0.018	**1.8**	0.264
Acetyllysine	**0.2**	0.016	**1.0**	0.902	**0.3**	0.186	**2.3**	0.060
Kynurenic acid	**0.6**	0.350	**0.5**	0.213	**2.3**	0.030	**0.7**	0.656
N-Acetyl-glutamate[1]	**1.0**	0.970	**2.5**	0.010	**1.0**	0.450	**0.8**	0.022
N-Acetyl-glutamate[2]	**0.9**	0.679	**1.3**	0.106	**0.7**	0.203	**0.5**	0.218

Citrate	**0.4**	0.173	**3.0**	0.007	**0.3**	0.001	**0.4**	0.205
2-Dehydro-D-gluconate	**ND**	_	**1.4**	0.720	**1.2**	0.328	**1.0**	0.991
D-Gluconate	**0.8**	0.558	**0.9**	0.890	**0.8**	0.851	**0.5**	0.535
D-Erythrose-4-P	**ND**	_	**ND**	_	**0.7**	0.101	**2.9**	0.005
Trp	**0.5**	0.190	**0.8**	0.485	**0.9**	0.505	**1.2**	0.698
D-Glucarate[1]	**0.7**	0.375	**1.5**	0.256	**0.8**	0.137	**0.9**	0.358
Deoxyribose-P	**1.1**	0.549	**1.2**	0.205	**1.0**	0.953	**1.0**	0.926
Prephenate	**ND**	_	**ND**	_	**1.3**	0.627	**1.8**	0.413
Deoxyuridine	**0.9**	0.456	**1.2**	0.483	**1.0**	0.869	**1.3**	0.335
Ribose-P	**2.1**	0.078	**3.8**	0.142	**1.5**	0.103	**1.4**	0.417
Thymidine	**0.3**	0.127	**1.6**	0.386	**ND**	_	**ND**	_
Ribose-P	**ND**	_	**ND**	_	**2.3**	0.149	**2.0**	0.191
Cytidine	**10.9**	0.070	**1.0**	0.885	**ND**	_	**ND**	_
Uridine	**0.6**	0.376	**ND**	_	**5.0**	0.010	**2.5**	0.576
Deoxyinosine	**1.5**	0.139	**0.4**	0.091	**0.5**	0.497	**2.4**	0.557
Shikimate-3-P	**13.0**	0.023	**1.8**	0.238	**2.0**	0.004	**0.4**	0.079
D-Gluconolactone-6-P	**ND**	_	**ND**	_	**0.8**	0.057	**1.0**	0.929
D-Glucosamine-1-P	**1.6**	0.058	**2.0**	0.009	**2.2**	0.053	**1.0**	0.805
D-Fructose 6-phosphate	**5.0**	0.127	**5.2**	0.076	**ND**	_	**ND**	_
Glucose-P	**5.0**	0.127	**ND**	_	**0.7**	0.239	**4.1**	0.195
S-Ribosylhomocysteine (SRH)	**1.6**	0.001	**1.0**	0.915	**3.7**	0.010	**1.4**	0.045
Adenosine	**1.3**	0.442	**1.9**	0.055	**1.5**	0.055	**1.0**	0.868
Inosine	**1.2**	0.653	**1.5**	0.351	**6.4**	0.071	**1.9**	0.173
6-Phosphogluconate	**ND**	_	**ND**	_	**0.7**	0.219	**1.1**	0.737
Xanthosine	**4.0**	0.002	**2.1**	0.063	**3.3**	0.018	**0.8**	0.526
D-Sedoheptulose-1,7-P	**1.2**	0.324	**1.6**	0.044	**ND**	_	**ND**	_
Argininosuccinate	**0.4**	0.009	**1.9**	0.306	**0.6**	0.274	**1.2**	0.568
N-Acetyl-glucosamine-1,6-P	**2.0**	0.026	**1.9**	0.030	**ND**	_	**ND**	_
Glutathione	**1.0**	0.991	**0.5**	0.473	**1.0**	0.897	**1.0**	0.995
dCMP	**1.3**	0.036	**1.7**	0.347	**1.2**	0.693	**0.3**	0.100
dUMP	**1.1**	0.843	**1.5**	0.208	**1.1**	0.721	**1.6**	0.181
Octoluse 8,1-P	**1.7**	0.000	**2.1**	0.061	**1.6**	0.261	**ND**	_
dTMP	**0.6**	0.361	**1.2**	0.631	**0.8**	0.196	**1.0**	0.954
CMP	**0.5**	0.016	**0.5**	0.028	**0.6**	0.063	**0.3**	0.008
UMP[1]	**0.6**	0.067	**2.0**	0.011	**0.6**	0.030	**1.5**	0.023
dAMP	**0.5**	0.265	**3.3**	0.107	**ND**	_	**ND**	_
Trehalose/Sucrose	**0.7**	0.017	**0.8**	0.051	**0.6**	0.173	**0.7**	0.259
Cellobiose	**ND**	_	**ND**	_	**1.0**	0.956	**1.1**	0.331
dGMP	**0.6**	0.203	**1.8**	0.111	**0.9**	0.623	**0.9**	0.766
IMP[2]	**ND**	_	**ND**	_	**ND**	_	**ND**	_
GMP	**0.4**	0.339	**2.7**	0.107	**0.4**	0.230	**1.0**	0.951
XMP	**ND**	_	**12.9**	0.372	**0.2**	0.461	**0.2**	0.424
Riboflavin	**0.4**	0.323	**0.7**	0.620	**0.6**	0.025	**0.6**	0.020
S-Adenosylhomocysteine (SAH)	**ND**	_	**ND**	_	**0.4**	0.080	**0.5**	0.144
dCDP	**ND**	_	**ND**	_	**0.7**	0.152	**1.1**	0.830
dTDP	**0.7**	0.588	**0.5**	0.421	**0.7**	0.072	**0.8**	0.353
CDP	**0.5**	0.208	**1.1**	0.679	**0.5**	0.113	**0.6**	0.136
UDP	**0.7**	0.404	**1.4**	0.385	**0.6**	0.054	**1.2**	0.753
Trehalose-6-P	**1.5**	0.281	**8.1**	0.002	**3.4**	0.066	**8.7**	0.080
Adenylyl sulfate	**1.3**	0.472	**1.2**	0.684	**1.8**	0.066	**2.3**	0.256
ADP	**0.9**	0.536	**1.0**	0.902	**0.8**	0.088	**0.9**	0.537
GDP	**1.0**	0.898	**1.6**	0.368	**0.5**	0.002	**0.4**	0.000

FMN	**0.7**	0.061	**1.7**	0.037	**0.7**	0.357	**0.9**	0.349
dCTP	**2.2**	0.146	**2.8**	0.212	**1.0**	0.863	**1.1**	0.750
dTTP	**1.2**	0.031	**1.2**	0.501	**1.1**	0.852	**1.0**	0.980
CTP	**1.5**	0.025	**3.3**	0.154	**0.7**	0.397	**0.6**	0.276
UTP	**2.0**	0.176	**4.3**	0.231	**1.0**	0.972	**0.7**	0.267
dATP	**1.5**	0.116	**0.9**	0.830	**0.9**	0.323	**1.0**	0.874
ATP	**1.6**	0.333	**2.0**	0.396	**1.0**	0.825	**0.9**	0.557
GTP	**4.4**	0.185	**5.8**	0.254	**1.0**	0.906	**0.5**	0.211
UDP-D-glucose	**3.0**	0.050	**14.0**	0.002	**0.5**	0.428	**5.0**	0.198
UDP-D-glucuronate	**ND**	-	**ND**	-	**0.2**	0.001	**0.8**	0.337
UDP-N-acetyl-glucosamine	**2.4**	0.031	**3.6**	0.014	**0.9**	0.703	**1.7**	0.219
Glutathione disulfide	**2.0**	0.200	**0.1**	0.001	**2.6**	0.047	**1.9**	0.646
NAD+	**0.9**	0.248	**1.0**	0.896	**1.0**	0.948	**1.0**	0.702
NADH	**0.9**	0.754	**0.5**	0.203	**0.8**	0.427	**0.3**	0.053
NADP+	**1.2**	0.441	**0.8**	0.291	**1.1**	0.108	**0.4**	0.126

Underlined p-values are <0.05.
[1, 2] Multiple species with identical m/z.
